# Phylogenomics supports microsporidia as the earliest diverging clade of sequenced fungi

**DOI:** 10.1186/1741-7007-10-47

**Published:** 2012-05-31

**Authors:** Salvador Capella-Gutiérrez, Marina Marcet-Houben, Toni Gabaldón

**Affiliations:** 1 Bioinformatics and Genomics Programme. Centre for Genomic Regulation (CRG) and UPF. Doctor Aiguader, 88. 08003 Barcelona, Spain

**Keywords:** Microsporidia, Fungi, Phylogenomics, Fungal Tree of Life

## Abstract

**Background:**

Microsporidia is one of the taxa that have experienced the most dramatic taxonomic reclassifications. Once thought to be among the earliest diverging eukaryotes, the fungal nature of this group of intracellular pathogens is now widely accepted. However, the specific position of microsporidia within the fungal tree of life is still debated. Due to the presence of accelerated evolutionary rates, phylogenetic analyses involving microsporidia are prone to methodological artifacts, such as long-branch attraction, especially when taxon sampling is limited.

**Results:**

Here we exploit the recent availability of six complete microsporidian genomes to re-assess the long-standing question of their phylogenetic position. We show that microsporidians have a similar low level of conservation of gene neighborhood with other groups of fungi when controlling for the confounding effects of recent segmental duplications. A combined analysis of thousands of gene trees supports a topology in which microsporidia is a sister group to all other sequenced fungi. Moreover, this topology received increased support when less informative trees were discarded. This position of microsporidia was also strongly supported based on the combined analysis of 53 concatenated genes, and was robust to filters controlling for rate heterogeneity, compositional bias, long branch attraction and heterotachy.

**Conclusions:**

Altogether, our data strongly support a scenario in which microsporidia is the earliest-diverging clade of sequenced fungi.

## Background

Microsporidia are spore-forming, intracellular eukaryotic parasites that infect a wide range of hosts [1]. Their taxonomic classification has evolved through time, representing one of the taxa for which the position in the tree of life has been subject to most radical changes [2]. Initially described as "yeast-like fungi" [3], microsporidians were soon re-assigned to Sporozoa, an assemblage of spore-forming protozoa. Later, microsporidia were proposed to be one of the most primitive eukaryotic lineages [4] based on initial electron microscopy studies, which suggested a remarkable absence of widespread eukaryotic features such as golgi bodies, peroxisomes, mitochondria and 9+2 microtubules. Indeed, in the context of the Archeozoa hypothesis [5], microsporidia were postulated to be direct descendants of a primitive eukaryote that predated mitochondrial endosymbiosis. This idea received support from initial phylogenies based on rRNA and elongation factor genes [6-8]. However, during the 1990s, a growing number of phylogenetic studies suggested a close relationship between microsporidia and fungi, and revealed that more basal positions of microsporidia were likely the result of long-branch attraction (LBA), a methodological artifact affecting highly divergent sequences [9]. Finally, the sequencing of *Encephalitozoon cuniculi *[10], and the discovery of microsporidian mitosomes [11], a relic version of mitochondria, precipitated the re-classification of microsporidia as fungi. This view is also supported by the presence of fungal traits, such as closed mitosis, spores containing chitin and trehalose [12,13], the presence of RPL21/RPS9 as neighboring genes [14], and the presence as independent genes of two domain pairs that are fused in non-fungal opisthokonts (ubiquitin/RPS30 and glutamyl/prolyl tRNA synthetases [15]).

While the fungal nature of microsporidia is now accepted, their exact position in the fungal tree remains debated [13,16]. Different analyses report alternative scenarios, none of which has been conclusively proven. For instance, an eight-gene analysis resolves microsporidia as a sister group to dikarya [17], while a six-loci analysis [18] clusters them with the chytrid *Rozella allomyces*. In the latter, this scenario was not significantly better supported than five alternative ones, including microsporidia being a sister-group to all fungi, to dikarya or to chytrids. Yet another placement, microsporidia as a sister group to zygomycota, was suggested from phylogenies of tubulins [19], but these divergent genes are prone to artifacts [20]. As molecular phylogenetics has been unable to satisfactorily resolve the issue, others have explored alternative molecular traits, such as the conservation of gene order. For instance, Lee *et al*. [14] found that microsporidia and zygomycotina bore similar gene arrangement in the *MAT *locus, while such organization was uncommon in other fungi. Based on this and other shared neighboring gene pairs, the authors tentatively placed microsporidia with zygomycota, but this interpretation has been recently challenged [21].

Most of these studies have been limited by the availability of a single microsporidian genome. This, together with the extremely fast-evolving nature of microsporidian sequences, has certainly limited the strength of previous conclusions. The recent release of five additional microsporidian genomes [22-25], as well as the higher availability of genomes from other early-branching fungi, allows us to re-assess the position of microsporidia with an unprecedented amount of information. In addition, higher genomic sampling among sister groups of fungi provides us now with a better choice of slow evolving out-groups. Increased taxonomic sampling is particularly important to tackle artifacts such as LBA [26,27], which is known to affect phylogenies of microsporidian sequences. Here, we set out to exploit all available genomic information to resolve the long-standing question of the phylogenetic position of microsporidia. Our analyses include assessment of gene order conservation, of thousands of individual gene phylogenies and of phylogenies based on 53 concatenated loci. In all cases, we contrasted alternative hypotheses in the presence of filters or models that control for potential sources of artifacts, such as compositional bias, LBA or heterotachy. Altogether, our data strongly support microsporidia as the earliest diverging clade of sequenced fungi.

## Results and discussion

### Chromosomal neighborhood conservation analysis

We first explored similarities in chromosomal gene order (also referred to as synteny or colinearity) between microsporidia and other clades, which has been used to associate microsporidia and zygomycotina [14]. This was mainly based on comparisons among *E. cuniculi*, *Rhizopus oryzae *and other fungi. However, as more microsporidian and zygomycota genomes became available it is necessary to assess the generality of such associations. For instance, a closer inspection at the MAT locus on a broader set of species and strains revealed that this locus had indeed undergone several re-arrangements [15]. Furthermore, a recent detailed analysis of the MAT locus found that this organization is likely to be either a derived ancestral opisthokont character or the result of convergence [21]. We believe that, besides this particular locus, the generally higher number of neighboring pairs found between *E. cuniculi *and *R. oryzae *must be considered carefully too, especially when relaxed criteria for synteny and homology are used. For instance, the occurrence of segmental duplications in only one of the lineages contributes to an overestimation of conserved pairs. Indeed, by analyzing the phylogenies of the putative syntenic pairs, we found that a significant number (at least 25%) of the proposed conserved neighboring pairs were related to gene expansions in *R. oryzae*, so that the same *E. cuniculi *pair would detect several *R. oryzae *pairs resulting from recent duplications (see Additional file 1, Figure S1). Considering that a recent whole genome duplication has been proposed for *R. oryzae *[28], it is necessary to account for this effect.

**Figure 1:**
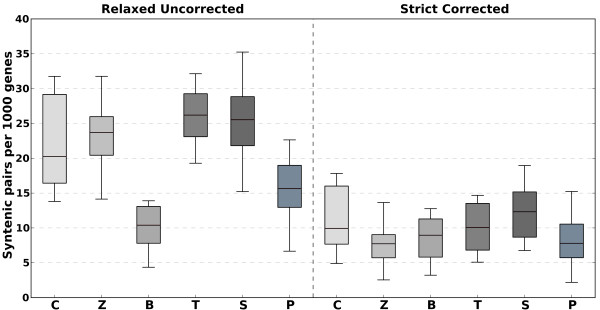
**Gene order conservation in all pair-wise comparisons between six microsporidian genomes and two chytrid, three zygomycotina, two basidiomycotina, one taphrinomycotina, two saccharomycotina, and two pezizomycotina from the primary set**. Results are shown for the two extreme syntenic pairs detection strategies:relaxed uncorrected, which is equivalent to that in [14] and a strict strategy correcting for segmental duplications. Exact counts are provided in additional file 1.

Here, we re-assess the level of synteny across microsporidians and other fungi, while controlling for the above mentioned effects. For this, we used the "relaxed synteny" approach described in [14] and a "strict synteny", which used orthology, rather than homology, relationships and stricter limits for re-arragements (see Methods). Our results (Figure 1, Additional file 1, Tables S1 and S2), indicated that apparent differences between groups are the result of the combined effect of differences in lineage-specific duplications and pools of shared homologs. When accounting for these differences, and when using a more stringent definition of synteny, we could not see any significant differences (*P*-value < 0.05, Kruskal-Wallis test, Figure 1). We conclude that there is a similarly low level of gene neighborhood conservation between microsporidia and other fungal groups. We thus turned to the analysis of sequence-based phylogenies to obtain better insights into their phylogenetic position.

### Microsporidian phylome analysis

**Figure 2:**
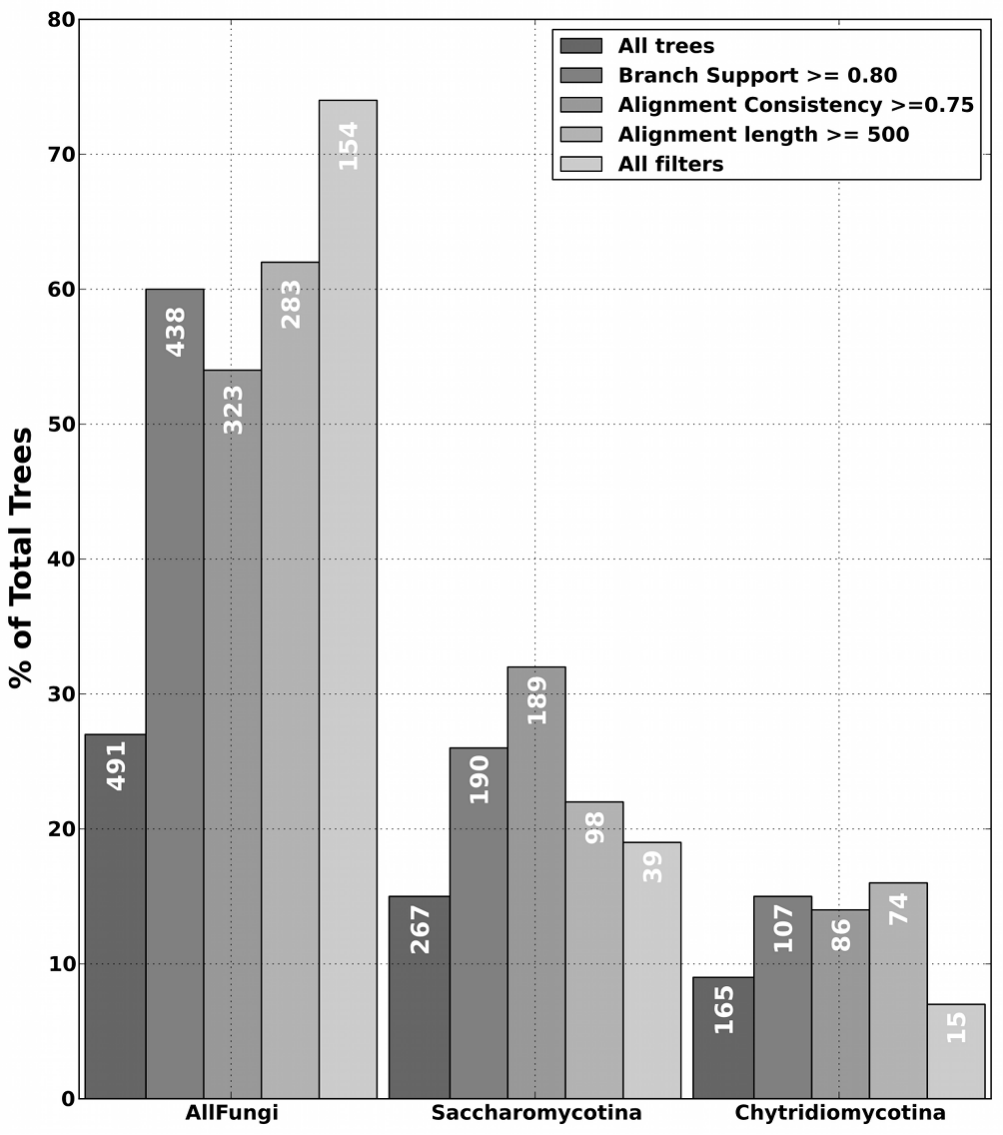
**Microsporidian sister-group analysis based on the six microsporidian phylomes**. Groups of bars represent the fraction of the phylomes that supports each scenario (only the three best supported are shown, see Additional file 1, Figures S2-S8 for the rest). Differently shadowed bars represent: from darker (left) to lighter (right) gray: all the trees, trees where the branch-support of the parental node of microsporidians and its sister group is higher than 0.8, trees where the alignment has a consistency score over 0.75, alignments with length larger than 500 columns, and the trees that pass all the previous filters.

Analyses of genome-wide collections of gene phylogenies (that is, phylomes) have been used to assess the support of alternative evolutionary scenarios [29,30], providing a broad view of the phylogenetic signal contributed by each individual gene, while identifying widespread trends that underlie the species phylogeny. We reconstructed a phylome for each of the six microsporidians, and scanned individual trees to define the sister-group of microsporidia (see Methods). Possibly due to a lack of sufficient phylogenetic signal, a large fraction (approximately 50%) of the trees did not recover a monophyly of the out-group species. As compared to other trees, these were based on shorter alignments and received lower support values. Manual inspection of some of these trees showed the presence of ancient duplications, highly divergent out-group sequences and other sources of phylogenetic artifacts. This is consistent with the previously reported prevalence of phylogenetic artifacts in individual microsporidian gene trees [16]. For the remaining trees, our analyses revealed a large topological diversity, with almost any possible scenario receiving some support (Figure 2, and Additional file 1, Figures S2-S8). However, an earliest branching position of microsporidia was consistently supported by the largest fraction of trees, receiving approximately three-fold higher support than the next ranked hypothesis. Notably, the scenario clustering microsporidia and zygomycotina [14] was ranked in the seventh position. Most remarkably, the support for the first scenario increased when using stringent filters for i) branch support, ii) alignment quality, iii) alignment length and iv) a combination of all three filters. This suggests that this topology is prevalent among genes carrying the highest levels of phylogenetic signal, and that a significant fraction of the alternative topologies may result from artifacts. Finally, we combined all individual gene trees into a super-tree by finding the species tree that implies the least number of total duplications in single-gene trees [31]. The resulting topology consistently supported the earliest branching position of microsporidia (Additional file 1, Figure S9). Altogether, these analyses show that, despite the generally low levels of phylogenetic signal, a consistent trend can be recognized that places microsporidia as a sister group to other fungi. This topology is much better supported than competing hypotheses, and the support for this hypothesis increases after applying filters that remove the least informative phylogenies.

### Gene concatenation analyses

**Figure 3:**
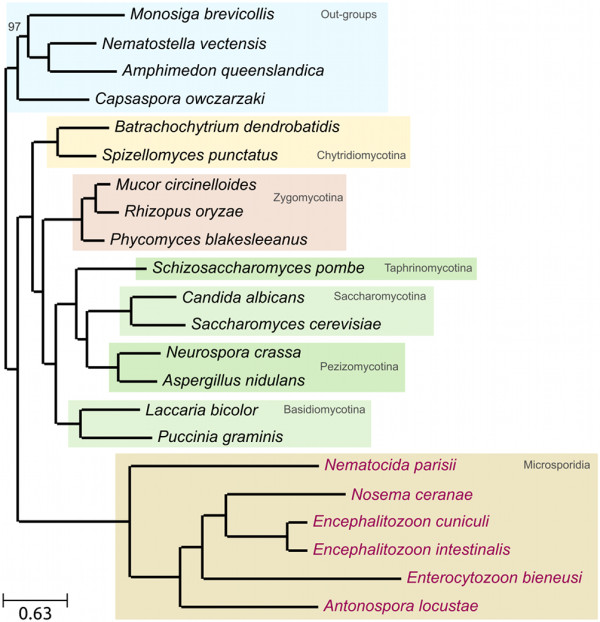
**ML tree derived from the concatenation of 53 widespread, single-copy proteins (see Additional file **1, **Table S6 for the list)**. The alignment was trimmed as explained in the Methods section to remove non-informative positions, resulting in 25,640 positions. The tree was derived using the LG evolutionary model. All aLRT-based support measures were 1.0. Bootstrap analysis was performed based on 100 alignment replicas, and single node with support below 100 is indicated. ML tree from the same alignment was also derived using the C40 CAT model, as implemented in PhyML. Additionally, to account for potential heterotachy, we derived a ML tree with a free rates parameter covarion model recently implemented in PhyML. Finally, a bayesian tree using PhyloBAYES v3.2 was inferred. All these analyses yielded identical topologies (see additional file 1).

The concatenation of multiple loci has been proposed to increase the level of phylogenetic signal and resolve difficult species relationships [32], and has been used extensively to reconstruct fungal phylogenies [30,33,34]. We adopted such strategy by concatenating 53 widespread proteins (see Methods). The resulting alignment was trimmed with trimAl v1.3 [35] to remove non-informative columns, and those containing gaps in all microsporidians, resulting in 25,640 positions. A second alignment of 9,745 positions was generated by removing potential compositional bias with BMGE [36]. Trees were derived from these alignments using Maximum Likelihood (ML) (LG and CAT) and Bayesian (CAT) analyses. Finally, all the above-mentioned alignments were re-coded into a reduced four-letters alphabet [37] and analyzed under a General Time Reversible (GTR) model to reduce any possible compositional bias. Irrespective of the phylogenetic method, evolutionary model or input alignment used, the resulting phylogenies are consistent with an earliest diverging position of microsporidia (see Figure 3 for ML (LG) and Additional file 1, Figures S10-S13 for the rest). Slight differences between the models attained the internal organization of microsporidia.

Next, we used ML analysis to contrast 12 competing scenarios (Additional file 1, Figure S14). The hypothesis placing microsporidia as a sister group to all other fungi was significantly more supported than any alternative scenario, as inferred from all but one of the eight statistical tests implemented in CONSEL [38]. The exception was the Shimodaira-Hasegawa test, which, as noted by the authors, is unreliable when more than two hypotheses are contrasted [38]. When repeating the test only contrasting these two hypotheses, the alternative one could be discarded.

**Figure 4:**
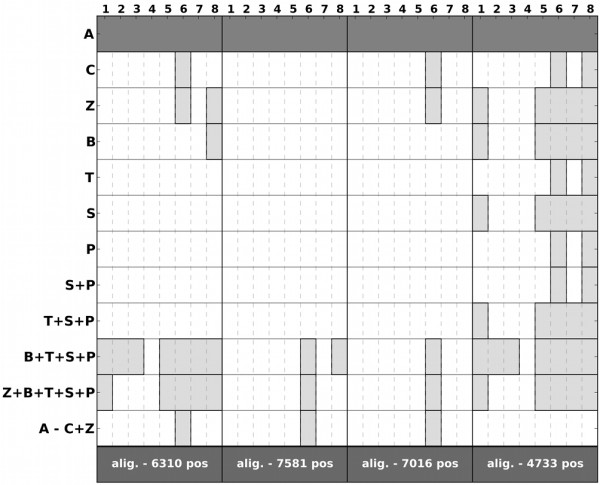
**ML analysis on a partitioned dataset, according to the number of residues variability among microsporidian sequences**. First partition groups 1 and 2 different residues, the second one contains columns with three different residues, the third one contains columns with four residues and the fourth group contains those columns with five or more different residues per column. The table below shows the results of the eight statistical tests implemented in CONSEL when comparing the support of each of the alternative topologies. Rows indicate the different alternative topologies considered (see Additional file 1, Figure S1): **A: **basal to all fungi, **C: **grouped with Chytrids, Z: grouped with Zygomycotina, **B: **grouped with Basidiomycotina, **T: **grouped with Taphrinomycotina, S: grouped with Saccharomycotina, **P: **grouped with Pezizomycotina, S+P: placed at the common ancestor of Saccharomycotina and Pezizomycotina, T+S+P: placed at the base of ascomycota, B+T+S+P: placed at the base of dikarya, B+T+S+P+Z: placed after Chytrids, A-C+Z: basal to fungi but Chytrids and Zygomycotina grouped. The columns represent the different statistical tests used: (1) Approximately Unbiased (AU) test, (2) Bootstrap probability (NP) test, (3) same as NP test, but calculated directly from the replicates (BP), (4) Bayesian posterior probability test calculated by the BIC approximation (PP), (5) the Kishino-Hasegawa (KH) test, (6) the Shimodaira-Hasegawa (SH) test, (7) the Weighted Kishino-Hasegawa (WKH) test, (8) the Weighted Shimodaira-Hasegawa (WSH) test. Dark grey represent the topology with the best likelihood, while light grey represent topologies that could not be discarded (*P*-value > = 0.05) by the specific test.

**Figure 5:**
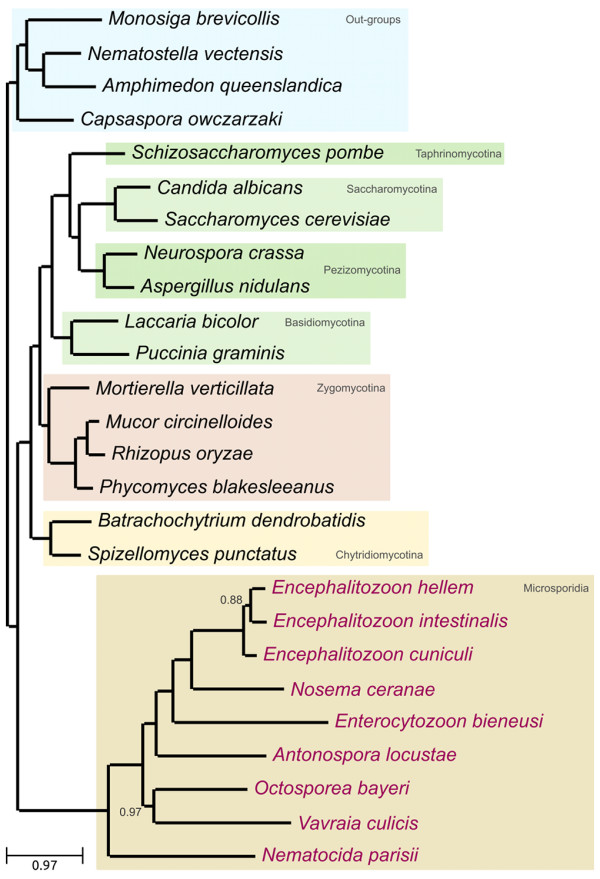
**ML species tree obtained from the concatenated alignment of 53 widespread, single-copy proteins (Additional file **1, **Table S6) extended with newly available three microsporidian species and one zygomycotina species (Additional file **1, **Table S5)**. The alignment was then trimmed to remove non-informative columns and columns that contained only gaps for the nine microsporidian species considered. The ML tree was reconstructed using LG as evolutionary model and SPR as tree topology search method as recommended in PhyML. A discrete gamma-distribution with four rate categories plus invariant positions was used, estimating the gamma parameter and the fraction of invariant positions from the data. Branch supports are SH-based aLRT statistics. Nodes with support below 1.0 are marked on the tree.

To examine the contribution of positions of varying evolutionary rates, we sequentially removed columns belonging to the 2, 4, 6, and 8 fastest rate categories out of 16 (see Methods). The resulting four sub-alignments thus contain a progressively lower number of fast-evolving positions and were used to repeat the tests performed above. Already, when removing the first two categories of fastest-evolving sites, the support for the alternative topology mentioned above disappeared completely (Additional file 1, Figure S15). In addition, we partitioned the alignment in four blocks differing in the level of divergence among microsporidian sequences. Again, the first scenario is the most supported in all partitions (Figure 4). However, the number of alternative hypotheses that cannot be rejected increases for the sub-alignments containing the least conserved residues. This result indicates that the proposed scenario is mostly supported by the slower evolving positions, whereas alternative topologies gain support in more saturated regions of the alignment. Thus all analyses consistently provide support for an early-branching position of microsporidia (Additional file 1, Table S3). The support for alternative hypotheses is always significantly lower and only increases when the underlying data are most likely to be affected by phylogenetic artifacts. Finally, to test the robustness of this topology to the addition of more taxa, we repeated the ML analyses on two expanded datasets, including additional species with complete genomes. In the first expanded dataset we increased the taxonomic sampling of microsporidians by adding genes from three recently released microsporidian genomes, which only became available during the preparation of this manuscript: namely *Octosporea bayeri*, *Encephalitozoon hellem*, and *Vavraia culicis *[39,40], and one additional Zygomycete: *Mortierella verticillata*. In the second expanded dataset, we included all species in the first expanded dataset plus 106 additional fungal species and 9 additional out-groups (138 species in total). The supported scenario remained robust and highly supported when these expanded datasets were used (see Figure 5 and Additional file 1, Figure S16).

LBA has been recognized as a major problem in phylogenies considering microsporidian sequences. We have adopted several strategies to minimize LBA as much as possible [27]. These include: i) shortening of problematic branches by means of a dense taxonomic sampling of microsporidia, ii) selection of multiple, slow evolving out-groups, iii) use of models accounting for LBA, compositional biases and heterotachy, and iv) removal of fastest evolving sites. After applying all these filters, our favored scenario remained highly supported. Yet, LBA may still be present to some degree. To explore its potential influence we performed additional tests. First, we repeated the analysis without the out-groups. Under these circumstances an alternative in-group topology, grouping chytrids and zygomycota, received similar support to an unaltered in-group topology. This suggests some effect of the out-group sequences which may be attributed to LBA. Nevertheless, our data mentioned above suggest that the position of microsporidia is stronger in the absence of factors promoting LBA. In addition, several other lines of evidence suggest that our preferred scenario is not the result of LBA. First, the position in which microsporidia are placed in our tree does not correspond to the expected one for extremely divergent sequences, as indicated by the inclusion of random sequences. Rather, extremely long branches are attracted within the out-groups, and not as a sister group to them, as found in many of the individual gene trees. This is also supported by a recommended test for LBA [27], in which microsporidian sequences in our concatenated alignments are substituted by random ones [27]. Under these conditions these extremely divergent sequences were mostly (80% of the cases in 100 replicas) within the out-groups, and only rarely (< 5%) in the position of microsporidia in our favored scenario. Second, and contrary to what would be expected under LBA, the support for our proposed scenario increases, rather than decreases, when applying stringent filters to the data. This is true for the analysis based on the concatenated alignment (removal of fast evolving sites) and those based on individual trees (use of longer, and less ambiguous alignments). Alternative scenarios gain some support only when more noisy subsets of the data are used, suggesting that existing biases erode rather than increase the support to our proposed scenario. Third, a parametric simulation [27] showed that the level of divergence present in our set was not sufficient to create an artifactual grouping of microsporidia and out-group sequences (see Methods). Thus, we conclude that our preferred scenario is likely not the result of LBA.

## Conclusion

Altogether, our results show that an earliest branching position of microsporidia is more supported than any alternative placement within fully-sequenced fungi. This conclusion is mostly based on sequence analysis, since low levels of gene order conservation provide insufficient information. Similarly, single-gene phylogenetic analyses are dominated by lack of phylogenetic signal and are prone to artifacts. However, our favored scenario appears to be supported by the largest fraction of genes, especially when loci likely to be carrying scarce phylogenetic signal are removed. Such scenario is also the most parsimonious in terms of the number of inferred duplications, and receives the strongest support in combined analyses of 53 loci. Our results are thus compatible with earlier studies supporting this association [20,41,42], as well as with a more recent, parallel analysis based on multiple Nematocida genomes (personal communication Christina Cuomo). This scenario has obvious taxonomic and evolutionary implications [16,18]. For instance, some traits shared with most but not all fungi, such as the absence of flagellated spores, would imply an independent, secondary loss in microsporidia. This and other characteristic traits of microsporidia, such as the presence of mitosomes, reduced genomes or the loss of cell-wall during the infective stage, would be the result of extreme adaptations to a parasitic lifestyle and, hence, provide little or none phylogenetic information. Taxonomically, our topology is consistent with microsporidia being defined as either the most basal lineage in fungi or as its closest sister-clade. Nevertheless, as discussed above, the fungal affiliation of microsporidia is supported by many shared traits (synapomorphies). In addition, since our taxonomic sampling is still reduced regarding basal groups we cannot test some hypotheses, such as the sisterhood of microsporidia and *Rozella *[18] or related species [43]. Interestingly, *Rozella *shares with microsporidia the absence of a cell wall during the infective stage, although it is unclear whether this would represent a convergent adaptation to an intracellular parasitic lifestyle. Of note, we found that the alternative placement of chytrids and zygomycota forming a monophyletic group, was often emerging among the alternative, less supported, hypotheses and that our analyses showed variations with respect to the branching order among microsporidians. Clearly, additional sequences will help to resolve these issues.

## Materials and methods

### Sequence data

Proteins encoded in 121 fungal genomes, including 6 microsporidians, as well as 4 out-group species were downloaded (Additional file 1, Table S4). This dataset was divided into two sub-sets: a primary set was formed by the 6 microsporidians, 4 out-groups, and 12 fungal species representing the main groups. A secondary set was formed by the remaining species (see Additional file 1, Table S4 for details). An expanded dataset with additional species (Additional file 1, Table S5) was created to test the robustness of the topology to increased taxonomic sampling.

### Analysis of gene order conservation

A similar analysis to that described in [14] was performed on the primary set. We used two approaches with varying levels of stringency. First, a "relaxed synteny" approach as described in [14] was applied. In brief, this method defines a pair of genes as "syntenic" if two genes in the query genome, that have at most three intervening genes, have homologs in the other genome separated by at most four genes. If the intervening genes in the query have homologs in the other genome, then these should be in a window of less than 15 genes. Second, we implemented a "strict synteny" method in which two genes in the same orientation separated by at most three intervening genes is considered to be syntenic if their orthologs (rather than simply homologs) in the other species are separated by at most three intervening genes. If one of the intervening genes has an ortholog in the other species, thus implying a genomic re-arrangement, then the pair is discarded. The Kruskal-Wallis test was used to assess for statistical significance of the differences [44].

### Phylome reconstruction and analyses

A phylome was reconstructed using as a seed each microsporidian species. Homologous were identified by Smith-Waterman [45] searches (E-value < 10^-5^, > 50% coverage). To maximize the number of gene trees encompassing all fungal groups, we adopted the following strategy: for each seed microsporidian sequence, homologs were searched in the primary set (see above). If no homolog was found for a given species, we extended the search to species in the secondary set belonging to the same group. Note that although a different microsporidian species is used as a seed in each phylome, they all contain homologs from all microsporidian species included in the analysis. Next, we reconstructed trees using the pipeline described in [46]. In brief, sequences are aligned with three different programs: MUSCLE v3.7 [47], MAFFT v6.712b [48], and DIALIGN-TX [49]. Alignments were run in forward and reverse direction (that is, using the Head or Tail approach [50]), and the six resulting alignments were combined with M-COFFEE [51], then the resulting meta-alignment was trimmed with trimAl v1.3 [35], (consistency-score cutoff 0.1667, gap-score cutoff 0.1). Trees were reconstructed using the best-fitting evolutionary model. Model selection was performed as follows: A Neighbor Joining (NJ) tree was reconstructed as implemented in BioNJ [52]; The likelihood of this topology was computed, with branch-length optimization, using seven evolutionary models (JTT, LG, WAG, Blosum62, MtREV, VT and Dayhoff), as implemented in PhyML v3.0 [53]; The two best-fitting models, as determined by the AIC criterion [54], were used to derive ML trees, using SPR (Subtree Pruning and Regrafting), a discrete gamma-distribution with four rate categories plus invariant positions, and the fraction of invariant positions and gamma parameter were inferred from the data. Branch support was computed using an aLRT (approximate likelihood ratio test) based on a chi-square distribution. Alignments and trees are available at ftp://ftp.cgenomics.org/microsporidia

We derived super-trees based on the whole collection of trees by using duptree [31] to search for the species topology implying the least number of duplications.

In order to determine the phylogenetic position of microsporidia as inferred from each individual gene tree, only trees with at least one representative from each of the six predefined groups (see Additional file 1. Table S4) were used. Trees were rooted using the most distant out-group sequence, and speciation and duplication events were detected using the species-overlap algorithm (species-overlap-score = 0.0) implemented in ETE v2 [55]. ETE was used to explore the species content of the nearest partition to the microsporidian sequences (that is, the sister-group). This defined the sister-group of microsporidia (for example, *Zygomycota *if only the zygomycota species were found, or *All fungi *if all the remaining fungal groups were sister to microsporidia).

### Gene concatenation analyses

We concatenated the trimmed alignments of 53 single-copy protein families with one-to-one orthologs in at least 18 species of the primary set. After removing positions with no residue variation in all sequences, and positions that only contained gaps in all microsporidia species, the alignment contained 25,640 positions. In addition, BMGE [36] was used to remove possible compositional heterogeneity bias across the sequences resulting in an alignment of 9,745 columns. Subsequently, four different tree reconstruction strategies were applied on these two alignments: First, ML trees were derived as described above, using LG [56], since this model best fit 50 out of 53 of the individual alignments. Second, ML trees were also derived using the C40 CAT model, as implemented in PhyML. Third, to account for potential heterotachy, we derived ML trees with a free rates parameter covarion model recently implemented in PhyML. Finally, Bayesian trees were inferred with PhyloBAYES v3.2 [57] using CAT, 2 independent Markov chain Monte Carlo (MCMC) chains, a saving frequency of 100 generations, and the following stop criteria, as recommended in the manual: 1) a maximum bipartition discrepancy (maxdiff) < 0.1 and 2) a minimum effective size > 100 for all parameters. Consensus trees were generated using the majority-rule.

To determine the contribution to the observed phylogenetic signal from positions with different evolutionary rates, we repeated the procedures mentioned above on sub-alignments resulting from sequentially removing fastest-evolving sites. TreePuzzle v5.2 [58] was used to assign residues to one of 16 possible rate categories. Then, the two, four, six and eight more divergent categories were subsequently removed from the two alignments described above. In addition, the alignment was partitioned into four sub-alignments based on the level of divergence within the microsporidia only. ML analysis was repeated on all resulting sub-alignments, as described above.

### Topological hypotheses testing

Using ETE [55], we constructed 12 alternative topologies (Additional file 1, Figure S14). To avoid influence of phylogenetic relationships not directly tested, internal microsporidian branches were collapsed and then resolved for each alignment with RAxML [59]. Branch lengths were optimized for each alignment and all competing hypotheses were compared using the eight statistical tests implemented in CONSEL [38]. The confidence set of topologies was obtained by collecting trees not significantly different (*P*-values > 0.05).

### Parametric test for LBA

We performed a parametric test as described in [27]. A total of 53 sequence families were simulated, as implemented in ROSE [60], using as a known tree one in which microsporidia clustered with chytrids. The same tree reconstruction procedures as explained before were implemented. In the reconstructed tree, microsporidia were not at the earliest branching position, indicating that LBA is not sufficient to artifactually result in that placement with the methodologies used (Additional file 1, Figure S17).

## Abbreviations

LBA: Long-branch attraction; ML: Maximum likelihood; BMGE: Block Mapping and Gathering with Entropy; NJ: Neighbor joining; aLRT: approximate likelihood ratio test; SPR: Subtree Pruning and Regrafting; GTR: General time-reversible; MCMC: Markov chain Monte Carlo.

## Competing interests

The authors declare that they have no competing interests.

## Authors' contributions

SCG performed all computations. SCG, MMH and TG analyzed the data. TG supervised the project and wrote the first draft of the manuscript. SCG and MMH participated in the writing of the manuscript.

## Supplementary Material

Additional file 1 **Supplementary tables and figures cited in the main text**. Legends can be found below.
**Additional file **1, **Figure S1**.
Showcase example to illustrate the confounding effects of recent segmental duplications in the detection of conserved syntenic pairs. The figure shows four syntenic pairs detected between the microsporidian Encephalitozoon cuniculi (code names in green) and the zygomycetes Rhizopus oryzae (code names in orange) using the "relaxed synteny" approach described in [14].
Relative locations in the genome are shown next to the relevant phylogenetic trees present in the reconstructed E. cuniculi phylome. Note that one of the genes was not included in the phylogenetic reconstruction because it did not pass the thresholds used. From the topology of the tree it is clear that the R. oryzae genes are paralogous to each other and that they result from a lineage-specific duplication that conserved the neighborhood of the genes. This leads to an over-estimation of the number of conserved syntenic pairs.
**Additional file **1, **Figure S2**
Analysis of the microsporidian sister groups for the phylome trees for all microsporidian phylomes where at least one member of each predefined group is present, and where out-group species are monophyletic. Groups of bars represent the percentage of trees that detect a given fungal group as sister to microsporidians. Differently colored bars represent the percentage of trees after applying filters focused on discarding trees that are more likely to present phylogenetic noise. From darker to lighter the bars represent: all the trees, trees where the branch-support of the node defining the association of microsporidians and their sister group is higher than 0.8, trees where the alignment has an average consistency score over 0.75, alignments with a length over 500 amino acids and the trees that pass all the filters.
**Additional file **1, **Figure S3**
Same as Additional file 1 Figure S3 but using only A. locusteae phylome.
**Additional file **1, **Figure S4**
Same as Additional file 1 Figure S3 but using only E. bieneusi phylome.
**Additional file **1, **Figure S5**
Same as Additional file 1 Figure S3 but using only E. cuniculi phylome.
**Additional file **1, **Figure S6**
Same as Additional file 1 Figure S3 but using only E. intestinalis phylome.
**Additional file **1, **Figure S7**
Same as Additional file 1 Figure S3 but using only N. ceranae phylome.
**Additional file **1, **Figure S8**
Same as Additional file 1 Figure S3 but using only N. parisii phylome.
**Additional file **1, **Figure S9**
Super-tree constructed using duptree [31]. The 3,768 trees reconstructed in the microsporidian phylomes, where at least one member of each predefined group is present, were used.
**Additional file **1, **Figure S10**
Species tree obtained from the concatenated alignment of 53 widespread, single-copy proteins (Additional file 1 Table S6). The alignment was then trimmed to remove non-informative columns and columns that contained gaps for the six microsporidian species considered. The maximum likelihood tree was reconstructed using the CAT40 evolutionary model and using the SPR tree topology search method as recommended in PhyML [53] manual. A discrete gamma-distribution with four rate categories plus invariant positions was used, estimating the gamma parameter and the fraction of invariant positions from the data. Branch supports are SH-based aLRT statistics. Nodes with support below 1 are marked on the tree.
**Additional file **1, **Figure S11**
Species tree obtained from the concatenated alignment of 53 widespread, single-copy proteins (Additional file 1 Table S6). The alignment was then trimmed to remove non-informative columns and columns that contained gaps for the six microsporidian species considered. A Bayesian analysis was performed using PhyloBAYES v3.2 [57] using CAT as the evolutionary model. The analysis was performed using 2 independent MCMC with a saving frequency of 100 generations and the following stop criteria: 1) a maximum discrepancy across the bi-partitions (maxdiff) less than 0.1 and 2) a minimum effective size of, at least, 100 points for each parameter in the program. Finally, a consensus tree was generated using the majority-consensus rule. Nodes with posterior probability below 1 are marked on the tree.
**Additional file **1, **Figure S12**
Species tree obtained from the concatenated alignment of 53 widespread, single-copy proteins (Additional file 1 Table S6). The alignment was then trimmed to remove non-informative columns and columns that contained gaps for the six microsporidian species considered. A ML tree, accounting for potential heterotachy, was derived with a free rates parameter covarion model recently implemented in PhyML (provided by S. Guindon). Nodes with support below 1 are marked on the tree.
**Additional file **1, **Figure S13**
Species tree obtained from the concatenated alignment of 53 widespread, single-copy proteins (Additional file 1 Table S6). The alignment was then trimmed to remove non-informative columns and columns that contained gaps for the six microsporidian species considered. Resulting alignment was recoded to a reduced four-letters alphabet [37]. A maximum likelihood tree was derived under a general time reversible (GTR) model as implemented in PhyML [53]. A discrete gamma-distribution with four rate categories plus invariant positions was used, estimating the gamma parameter and the fraction of invariant positions from the data. Branch supports are SH-based aLRT statistics. Nodes with support below 1 are marked on the tree.
**Additional file **1, **Figure S14**
Alternative species tree topologies used for statistical comparisons to the ML topology (Figure 3 in the main paper). All alternative topologies were generated with ETE [55]. Microsporidia species were collapsed in the initial topologies to avoid favoring any internal organization. ML reconstruction on the complete alignment was performed in two steps, the first determines internal organization of microsporidia using RaxML [59] while the second optimizes tree branch lengths to compute the likelihood for alternative scenarios. Different alternative topologies considered for microsporidia position were: **A) **basal to all fungi, **C) **grouped with Chytridiomycotina, **Z) **grouped with Zygomycotina, **B) **grouped with Basidiomycotina, **S) **grouped with Saccharomycotina, **P) **grouped with Pezizomycotina, **T) **grouped with Taphrinomycotina, S+P) placed at the common ancestor of Saccharomycotina and Pezizomycotina, T+S+P) placed at the base of ascomycotina, B+T+S+P) placed at the base of dykarya, Z+B+T+S+P) placed at the common ancestor of dykarya and Zygomycotina, A - C+Z) basal to all fungi but grouping Zygomycotina and Chytridiomycotina.
**Additional file **1, **Figure S15**
Summary of the results of eight statistical tests comparing twelve alternative species tree topologies (see Additional file 1 Figure S14). These tests were performed on on four partitions of the 53 proteins concatenated alignment (Additional file 1 Table S64). The alignment was trimmed to remove non-informative columns and columns that contained gaps for the six microsporidian species considered. Then, partitions were generated by sequentially removing the 2, 4, 6 and 8 fastest-evolving sites categories, as classified by TreePuzzle v5.2 [58]. Finally the alternative topologies were tested on each separate partition. Dark gray indicates the topology with the best likelihood, while light gray indicate the topologies whose likelihood is not significantly different to the best one, according to a given test. White squares represent those tree topologies that can be confidently rejected according to a given test.
**Additional file **1, **Figure S16**
Species tree obtained from the concatenated alignment of 42 widespread, single-copy proteins including all species used on this study (Additional file 1 Tables 4 and 5). Original 53 genes (Additional file 1 Table S6) were used to search for single-copy orthologs in all species and those present in few species were removed from the concatenated alignment. The resulting alignment was then trimmed to remove non-informative columns and columns that contained gaps for the nine microsporidian species considered. ML tree was reconstructed using LG evolutionary model, since it was the best fitting evolutionary model in 39 out of 42 final selected genes, and using SPR as tree topology search method such as recommended in PhyML [53] manual. A discrete gamma-distribution with four rate categories plus invariant positions was used, estimating the gamma parameter and the fraction of invariant positions from the data. Branch supports are SH-based aLRT statistics. Nodes with support below 1 are marked on the tree.
**Additional file **1, **Figure S17**
Topology of the tree used as input in the simulations with ROSE [60] (A) and the final tree inferred applying the standard procedure of concatenation and maximum likelihood reconstruction (B).
**Additional file **1, **Table S1**
The number of syntenic pairs as detected with the "relaxed synteny" method (see main text). Columns represent each of the microsporidian genomes. Rows represent the species against which a given microsporidian genome is compared. For each pair of genomes the following information is available: the first column for each microsporidian species represents the total number of shared homologs in these two species, the second column represents the number of syntenic pairs found, without any correction, the third column represents the normalized number of pairs per 1000 shared homologs. The fourth and fifth columns represent the same data as the second and third column but correcting the values by counting paralogous pairs only once (see main text).
**Additional file **1, **Table S2**
Table representing the number of pairs of proteins with conserved synteny in two genomes detected with the strict method (see main text). Numbers are normalized in this case by the number of shared orthologs (shared pairs per 1000 shared orthologs). The table follows the same structure as Additional file 1 Table S1.
**Additional file **1, **Table S3**
Summary of the analysis run in this paper and the main conclusions extracted for each analysis. Table is read from left to right, with each subsequent analysis acting on the one located to its left. The last column indicates the main conclusion obtained for a given analysis.
**Additional file **1, **Table S4**
List of species included in the analysis. Columns indicate, in this order, the fungal group, the three letter code used throughout the analysis, the species name, as found in the download site, the source where the proteomes were downloaded, and date when the data were acquired. Species belonging to the primary set are shadowed, all the rest belong to the secondary set.
**Additional file **1, **Table S5**
List of new species included in the concatenation analyses. Columns indicate, in this order, the taxonomic group, the species code used throughout the analysis, the species name, as found in the download site, the source where proteomes were downloaded, and date when the data were acquired.
**Additional file **1, **Table S6**
List of 53 widespread, single-copy proteins used in the concatenation. The E. cuniculi orthologs are listed. Columns represent the UNIPROT accession code, the gene name, the length of the protein and the description of the gene. Click here for file
