# Supplementary material for: Phylogenomics supports microsporidia as the earliest diverging clade of sequenced fungi
Source: BMC Biol. 2012 May 31;10:47. doi: 10.1186/1741-7007-10-47 (PMC3586952; doi:10.1186/1741-7007-10-47)
Supplement: Additional file 1 — Supplementary tables and figures cited in the main text. Legends can be found below. Additional file 1, Figure S1. Showcase example to illustrate the confounding effects of recent segmental duplications in the detection of conserved syntenic pairs. The figure shows four syntenic pairs detected between the microsporidian Encephalitozoon cuniculi (code names in green) and the zygomycetes Rhizopus oryzae (code names in orange) using the "relaxed synteny" approach described in [14]. Relative locations in the genome are shown next to the relevant phylogenetic trees present in the reconstructed E. cuniculi phylome. Note that one of the genes was not included in the phylogenetic reconstruction because it did not pass the thresholds used. From the topology of the tree it is clear that the R. oryzae genes are paralogous to each other and that they result from a lineage-specific duplication that conserved the neighborhood of the genes. This leads to an over-estimation of the number of conserved syntenic pairs. Additional file 1, Figure S2 Analysis of the microsporidian sister groups for the phylome trees for all microsporidian phylomes where at least one member of each predefined group is present, and where out-group species are monophyletic. Groups of bars represent the percentage of trees that detect a given fungal group as sister to microsporidians. Differently colored bars represent the percentage of trees after applying filters focused on discarding trees that are more likely to present phylogenetic noise. From darker to lighter the bars represent: all the trees, trees where the branch-support of the node defining the association of microsporidians and their sister group is higher than 0.8, trees where the alignment has an average consistency score over 0.75, alignments with a length over 500 amino acids and the trees that pass all the filters. Additional file 1, Figure S3 Same as Additional file 1 Figure S3 but using only A. locusteae phylome. Additional file 1, Figure [file 1741-7007-10-47-S1.PDF]

Supplementary material for the article:

# “Phylogenomics supports microsporidia as the earliest branching group among sequenced fungi”

Salvador Capella-Gutierrez, Marina Marcet-Houben and Toni Gabaldón*

## Figure S1

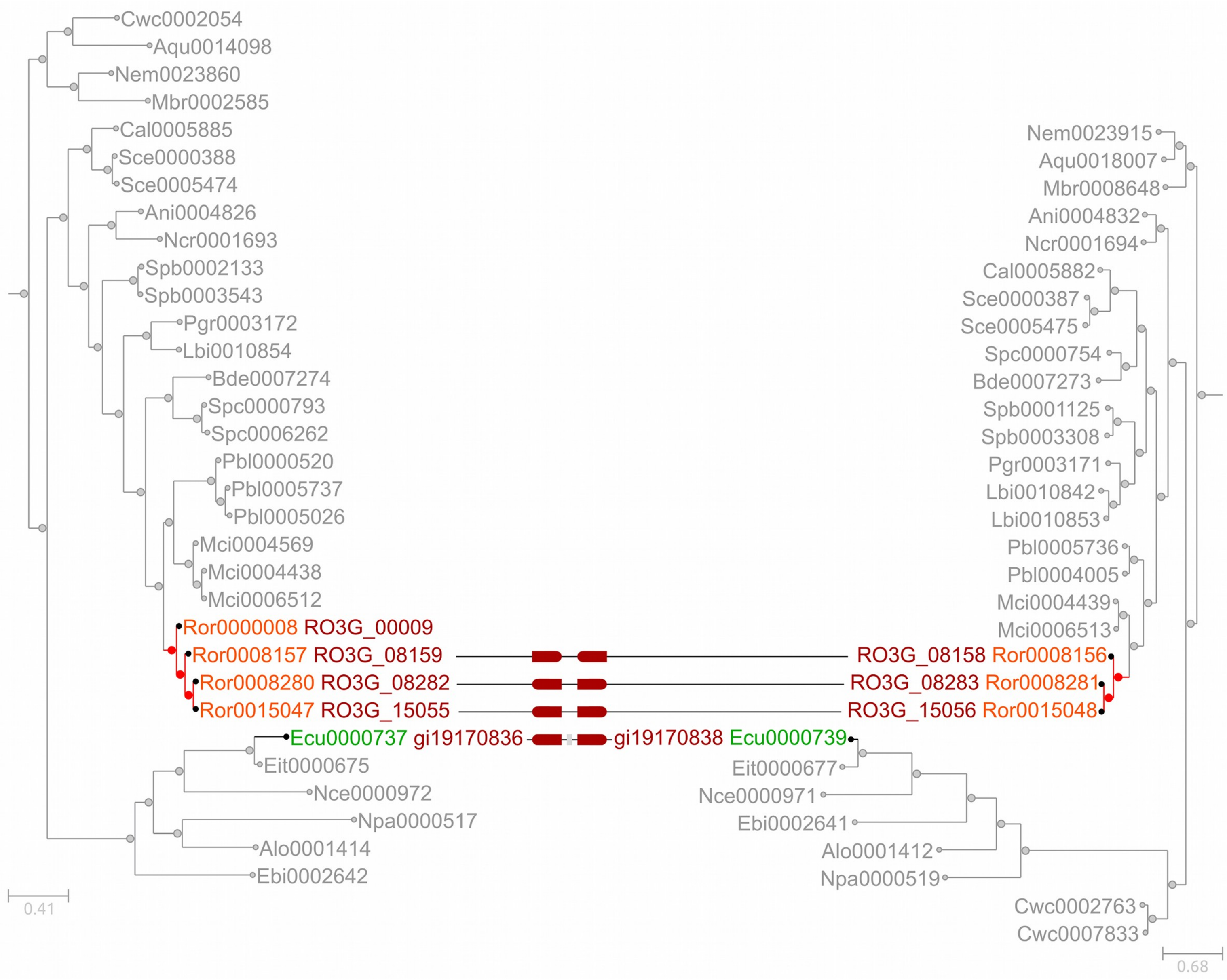

Cwc0002054
Aqu0014098
Nem0023860
Mbr0002585
Cal0005885
Sce0000388
Sce0005474
Ani0004826
Ncr0001693
Spb0002133
Spb0003543
Pgr0003172
Lbi0010854
Bde0007274
Spc0000793
Spc0006262
Pbl0000520
Pbl0005737
Pbl0005026
Mci0004569
Mci0004438
Mci0006512
Ror0000008 RO3G_00009
Ror0008157 RO3G_08159
Ror0008280 RO3G_08282
Ror0015047 RO3G_15055
Ecu0000737 gi19170836
Eit0000675
Nce0000972
Npa0000517
Alo0001414
Ebi0002642
0.41
Nem0023915
Aqu0018007
Mbr0008648
Ani0004832
Ncr0001694
Cal0005882
Sce0000387
Sce0005475
Spc0000754
Bde0007273
Spb0001125
Spb0003308
Pgr0003171
Lbi0010842
Lbi0010853
Pbl0005736
Pbl0004005
Mci0004439
Mci0006513
RO3G_08158 Ror0008156
RO3G_08283 Ror0008281
RO3G_15056 Ror0015048
gi19170838 Ecu0000739
Eit0000677
Nce0000971
Ebi0002641
Alo0001412
Npa0000519
Cwc0002763
Cwc0007833
0.68

# Figure S2

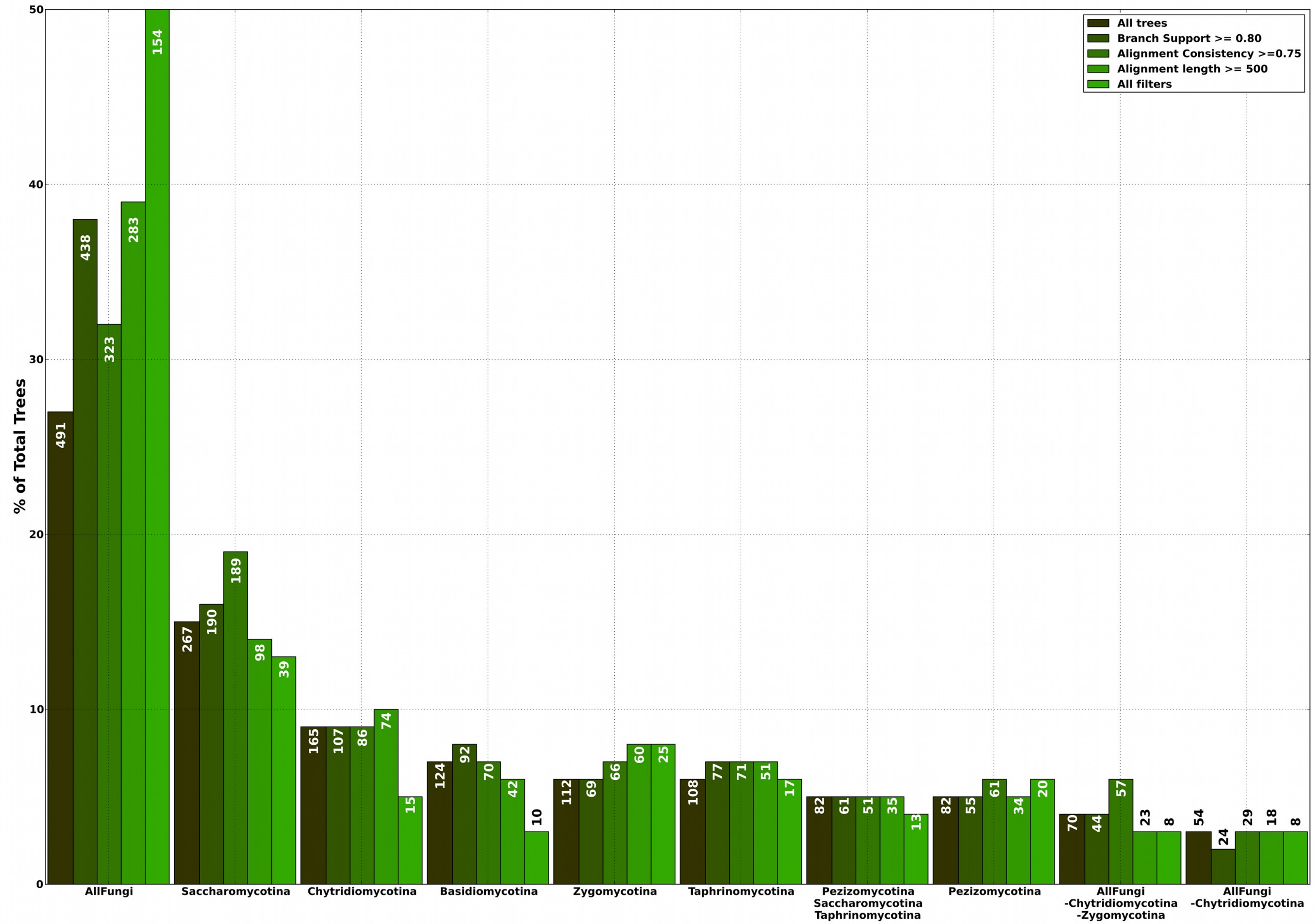

All trees
Branch Support >= 0.80
Alignment Consistency >=0.75
Alignment length >= 500
All filters
% of Total Trees
0
10
20
30
40
50
491
438
323
283
154
267
190
189
98
39
165
107
86
74
15
124
92
70
42
10
112
69
66
60
25
108
77
71
51
17
82
61
51
35
13
82
55
61
34
20
70
44
57
23
8
54
24
29
18
8
AllFungi
Saccharomycotina
Chytridiomycotina
Basidiomycotina
Zygomycotina
Taphrinomycotina
Pezizomycotina
Saccharomycotina
Taphrinomycotina
Pezizomycotina
AllFungi
-Chytridiomycotina
-Zygomycotina
AllFungi
-Chytridiomycotina

# Figure S3

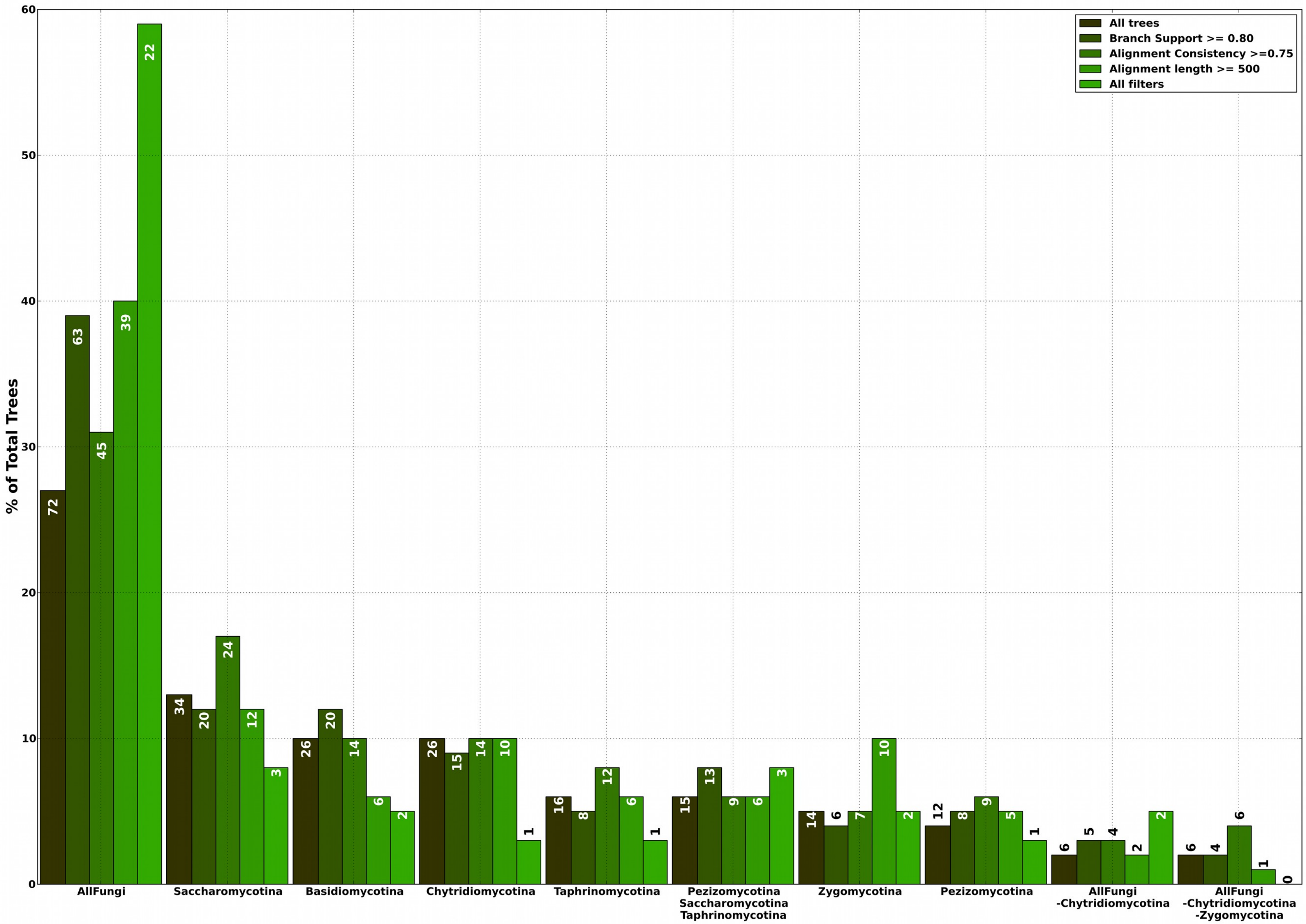

% of Total Trees
0
10
20
30
40
50
60
All trees
Branch Support >= 0.80
Alignment Consistency >=0.75
Alignment length >= 500
All filters
72 63 45 39 22
34 20 24 12 3
26 20 14 6 2
26 15 14 10 1
16 8 12 6 1
15 13 9 6 3
14 6 7 10 2
12 8 9 5 1
6 5 4 2 2
6 4 6 1 0
AllFungi
Saccharomycotina
Basidiomycotina
Chytridiomycotina
Taphrinomycotina
Pezizomycotina Saccharomycotina Taphrinomycotina
Zygomycotina
Pezizomycotina
AllFungi -Chytridiomycotina
AllFungi -Chytridiomycotina -Zygomycotina

# Figure S4

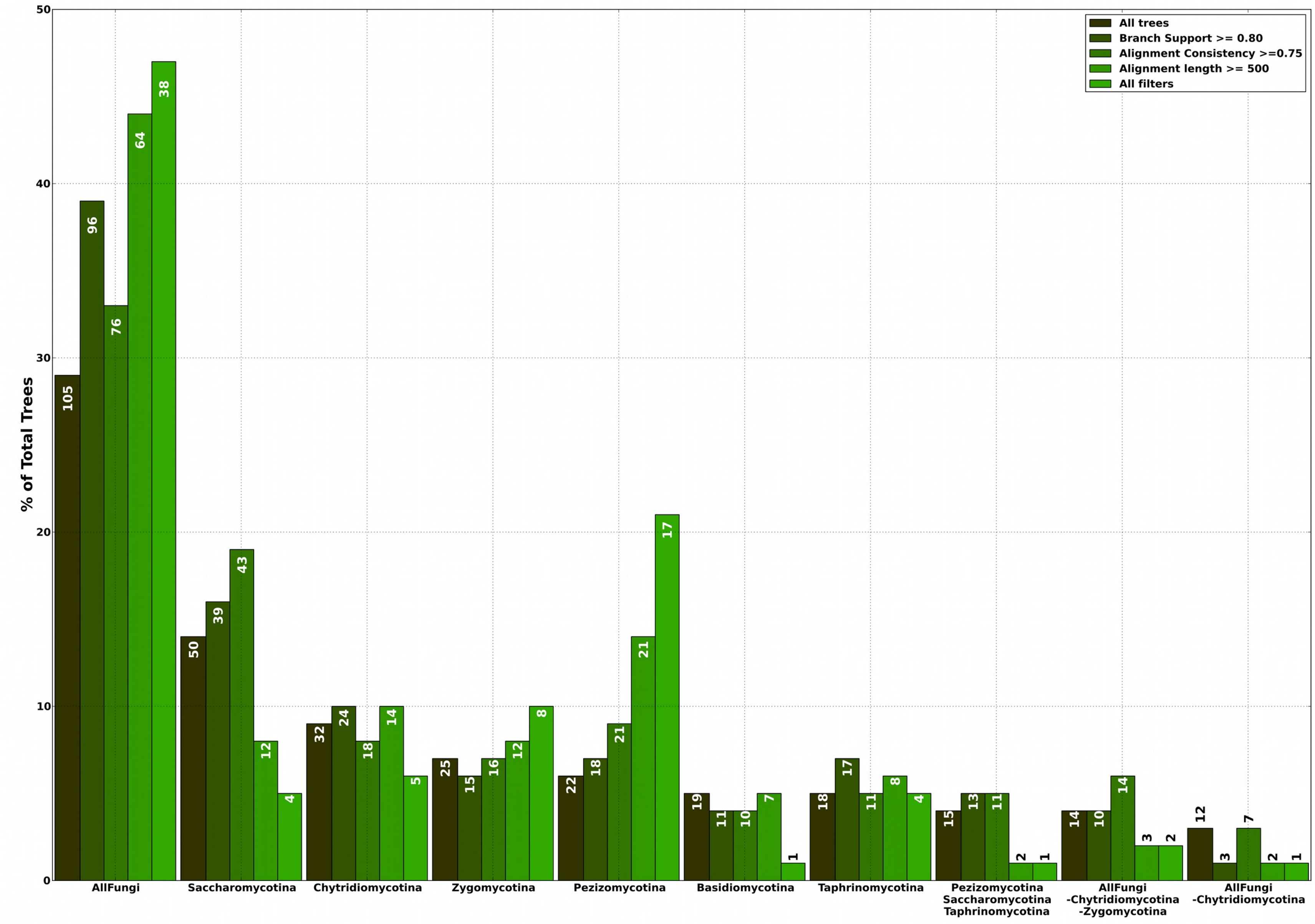

% of Total Trees
0
10
20
30
40
50
All trees
Branch Support >= 0.80
Alignment Consistency >=0.75
Alignment length >= 500
All filters
105
96
76
64
38
50
39
43
12
4
32
24
18
14
5
25
15
16
12
8
22
18
21
21
17
19
11
10
7
1
18
17
11
8
4
15
13
11
2
1
14
10
14
3
2
12
3
7
2
1
AllFungi
Saccharomycotina
Chytridiomycotina
Zygomycotina
Pezizomycotina
Basidiomycotina
Taphrinomycotina
Pezizomycotina
Saccharomycotina
Taphrinomycotina
AllFungi
-Chytridiomycotina
-Zygomycotina
AllFungi
-Chytridiomycotina

# Figure S5

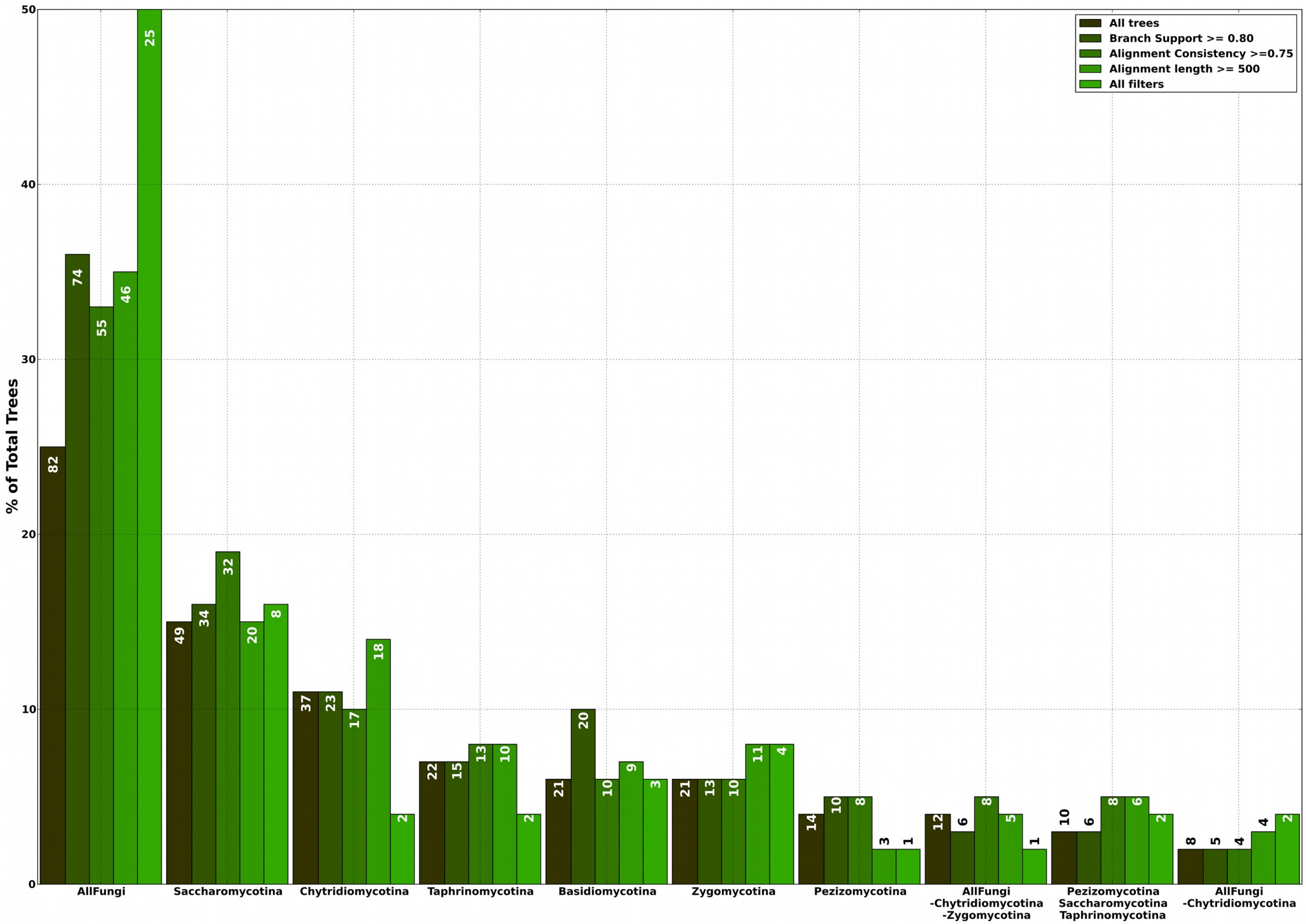

All trees
Branch Support >= 0.80
Alignment Consistency >=0.75
Alignment length >= 500
All filters
% of Total Trees
0
10
20
30
40
50
AllFungi
Saccharomycotina
Chytridiomycotina
Taphrinomycotina
Basidiomycotina
Zygomycotina
Pezizomycotina
AllFungi -Chytridiomycotina -Zygomycotina
Pezizomycotina Saccharomycotina Taphrinomycotina
AllFungi -Chytridiomycotina
82 74 55 46 25
49 34 32 20 8
37 23 17 18 2
22 15 13 10 2
21 20 10 9 3
21 13 10 11 4
14 10 8 3 1
12 6 8 5 1
10 6 8 6 2
8 5 4 4 2

# Figure S6

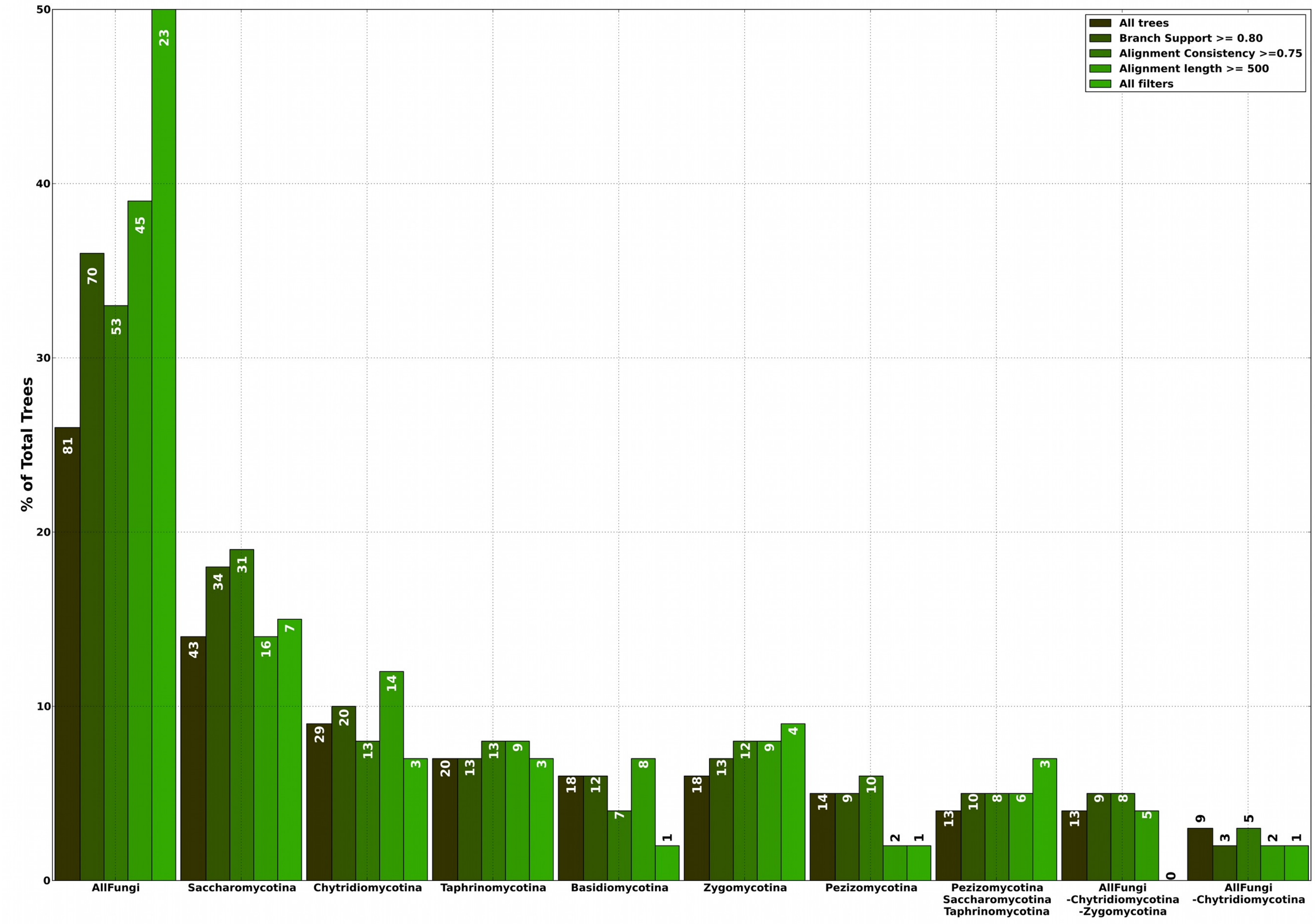

% of Total Trees
0
10
20
30
40
50
All trees
Branch Support >= 0.80
Alignment Consistency >=0.75
Alignment length >= 500
All filters
81
70
53
45
23
43
34
31
16
7
29
20
13
14
3
20
13
13
9
3
18
12
7
8
1
18
13
12
9
4
14
9
10
2
1
13
10
8
6
3
13
9
8
5
0
9
3
5
2
1
AllFungi
Saccharomycotina
Chytridiomycotina
Taphrinomycotina
Basidiomycotina
Zygomycotina
Pezizomycotina
Pezizomycotina
Saccharomycotina
Taphrinomycotina
AllFungi
-Chytridiomycotina
-Zygomycotina
AllFungi
-Chytridiomycotina

# Figure S7

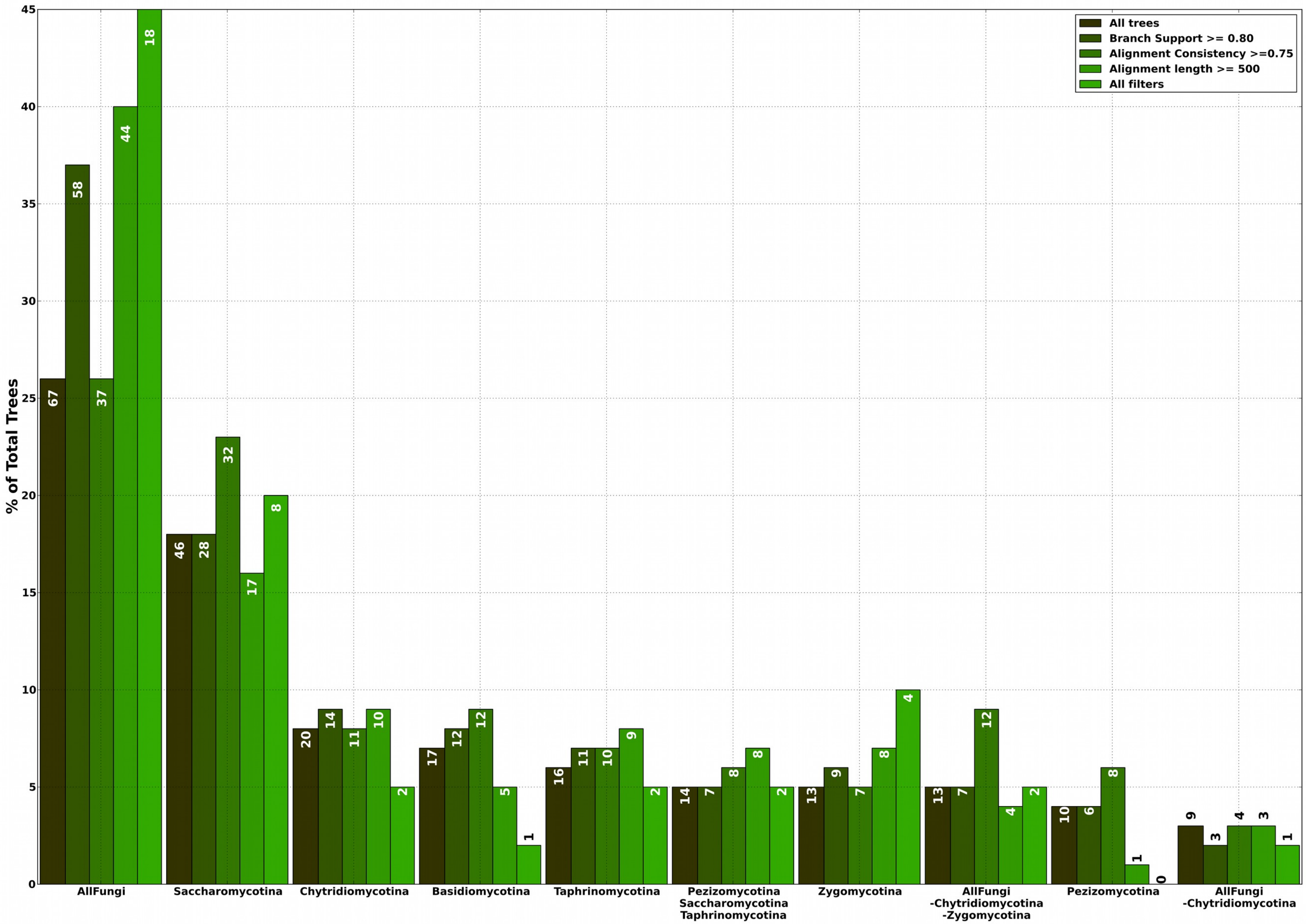

% of Total Trees
0
5
10
15
20
25
30
35
40
45
All trees
Branch Support >= 0.80
Alignment Consistency >=0.75
Alignment length >= 500
All filters
AllFungi
67 58 37 44 18
Saccharomycotina
46 28 32 17 8
Chytridiomycotina
20 14 11 10 2
Basidiomycotina
17 12 12 5 1
Taphrinomycotina
16 11 10 9 2
Pezizomycotina Saccharomycotina Taphrinomycotina
14 7 8 8 2
Zygomycotina
13 9 7 8 4
AllFungi -Chytridiomycotina -Zygomycotina
13 7 12 4 2
Pezizomycotina
10 6 8 1 0
AllFungi -Chytridiomycotina
9 3 4 3 1

# Figure S8

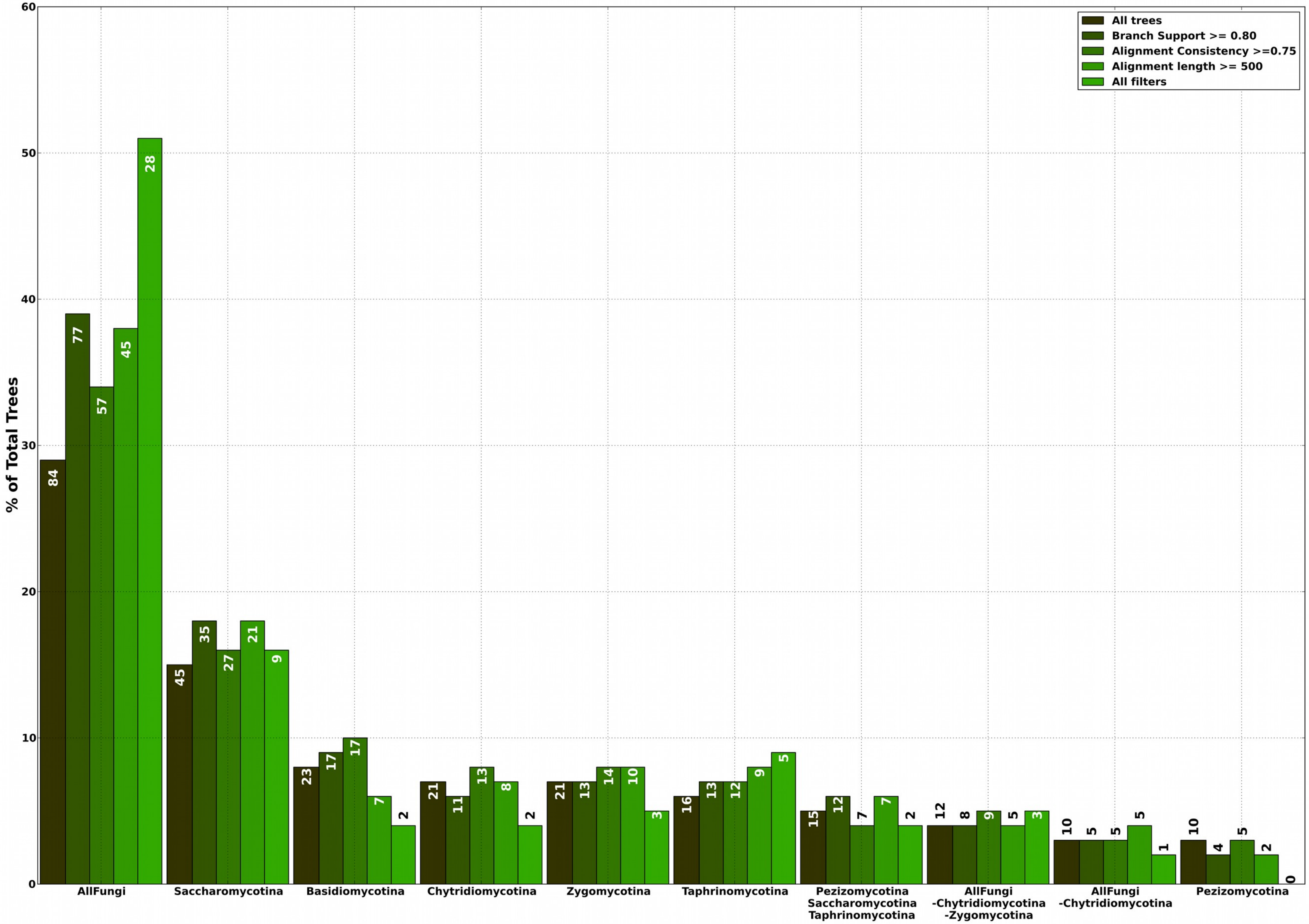

All trees
Branch Support >= 0.80
Alignment Consistency >=0.75
Alignment length >= 500
All filters
% of Total Trees
0
10
20
30
40
50
60
84
77
57
45
28
45
35
27
21
9
23
17
17
7
2
21
11
13
8
2
21
13
14
10
3
16
13
12
9
5
15
12
7
7
2
12
8
9
5
3
10
5
5
5
1
10
4
5
2
0
AllFungi
Saccharomycotina
Basidiomycotina
Chytridiomycotina
Zygomycotina
Taphrinomycotina
Pezizomycotina
Saccharomycotina
Taphrinomycotina
AllFungi
-Chytridiomycotina
-Zygomycotina
AllFungi
-Chytridiomycotina
Pezizomycotina

## Figure S9

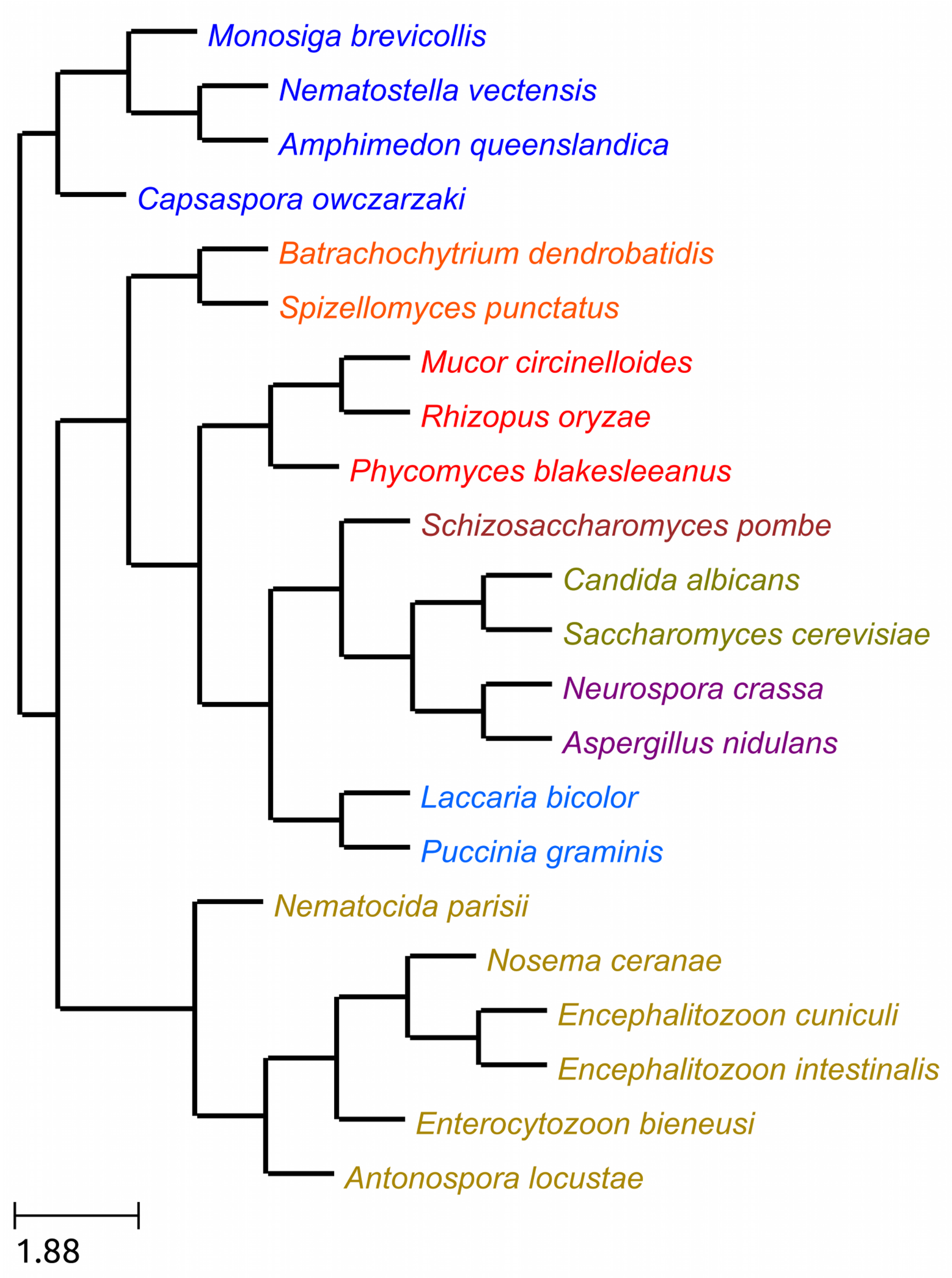

Monosiga brevicollis
Nematostella vectensis
Amphimedon queenslandica
Capsaspora owczarzaki
Batrachochytrium dendrobatidis
Spizellomyces punctatus
Mucor circinelloides
Rhizopus oryzae
Phycomyces blakesleeanus
Schizosaccharomyces pombe
Candida albicans
Saccharomyces cerevisiae
Neurospora crassa
Aspergillus nidulans
Laccaria bicolor
Puccinia graminis
Nematocida parisii
Nosema ceranae
Encephalitozoon cuniculi
Encephalitozoon intestinalis
Enterocytozoon bieneusi
Antonospora locustae
1.88

**Figure S10**

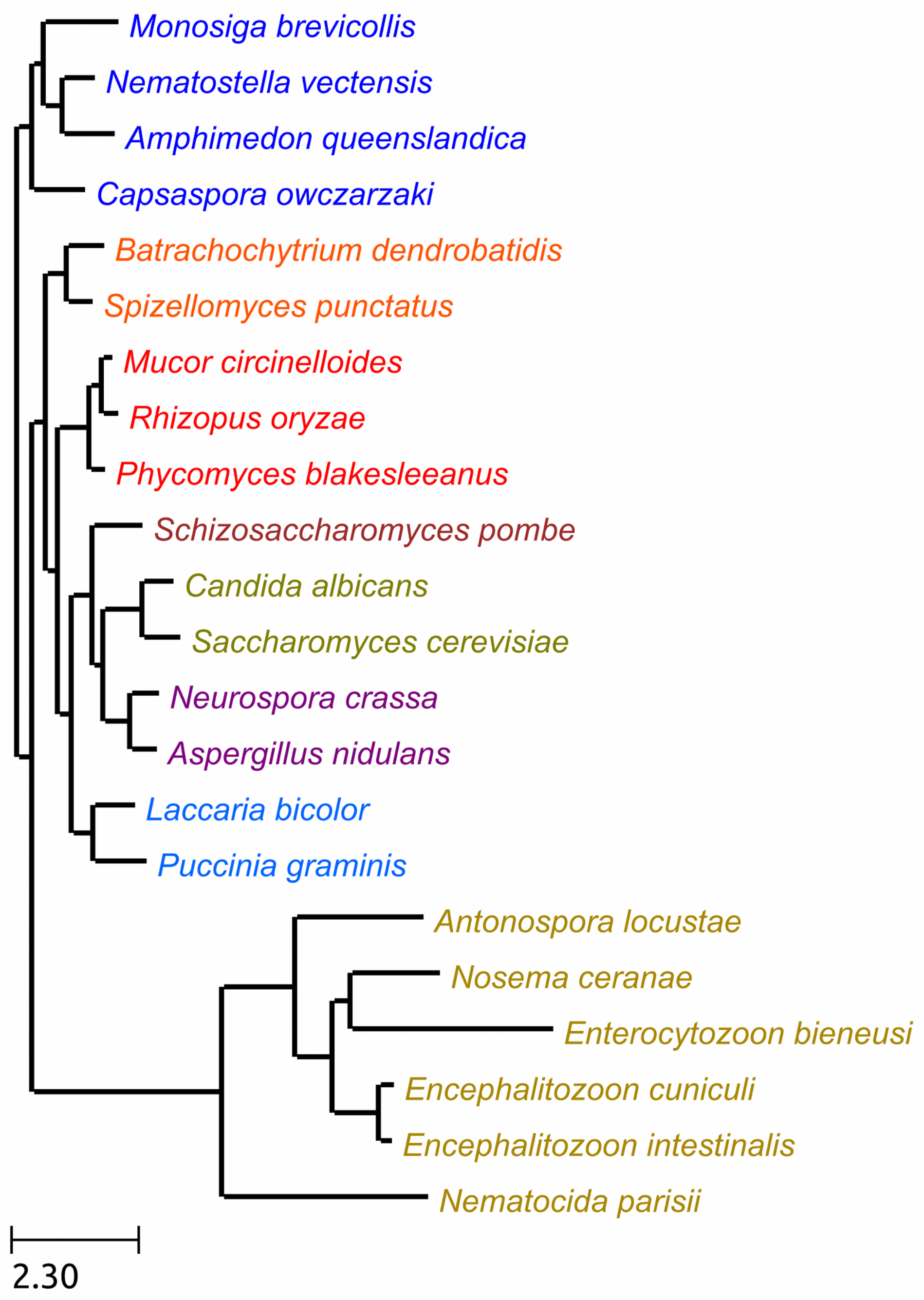
Monosiga brevicollis
Nematostella vectensis
Amphimedon queenslandica
Capsaspora owczarzaki
Batrachochytrium dendrobatidis
Spizellomyces punctatus
Mucor circinelloides
Rhizopus oryzae
Phycomyces blakesleeanus
Schizosaccharomyces pombe
Candida albicans
Saccharomyces cerevisiae
Neurospora crassa
Aspergillus nidulans
Laccaria bicolor
Puccinia graminis
Antonospora locustae
Nosema ceranae
Enterocytozoon bieneusi
Encephalitozoon cuniculi
Encephalitozoon intestinalis
Nematocida parisii
2.30

## Figure S11

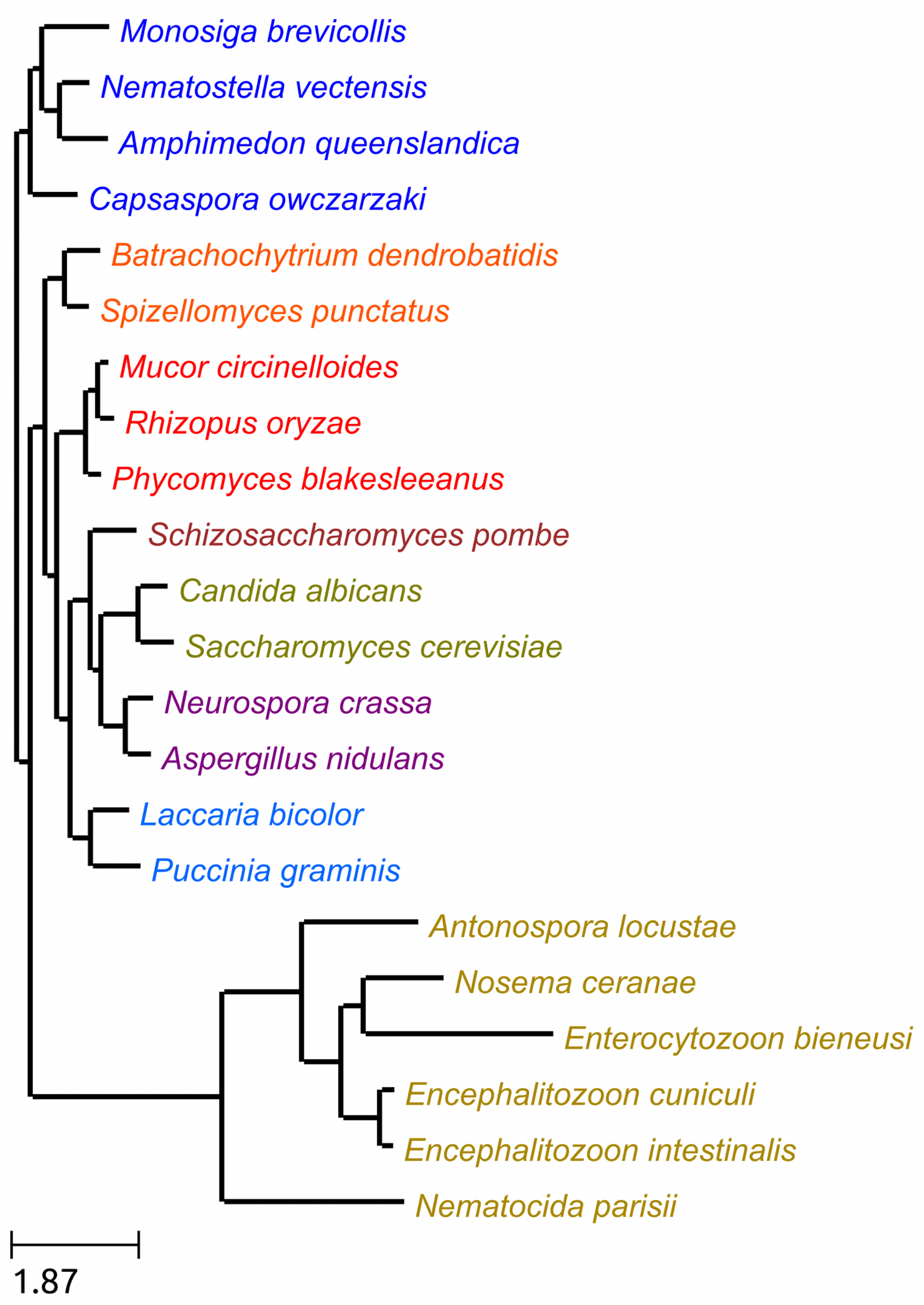
Monosiga brevicollis
Nematostella vectensis
Amphimedon queenslandica
Capsaspora owczarzaki
Batrachochytrium dendrobatidis
Spizellomyces punctatus
Mucor circinelloides
Rhizopus oryzae
Phycomyces blakesleeanus
Schizosaccharomyces pombe
Candida albicans
Saccharomyces cerevisiae
Neurospora crassa
Aspergillus nidulans
Laccaria bicolor
Puccinia graminis
Antonospora locustae
Nosema ceranae
Enterocytozoon bieneusi
Encephalitozoon cuniculi
Encephalitozoon intestinalis
Nematocida parisii
1.87

**Figure S12**

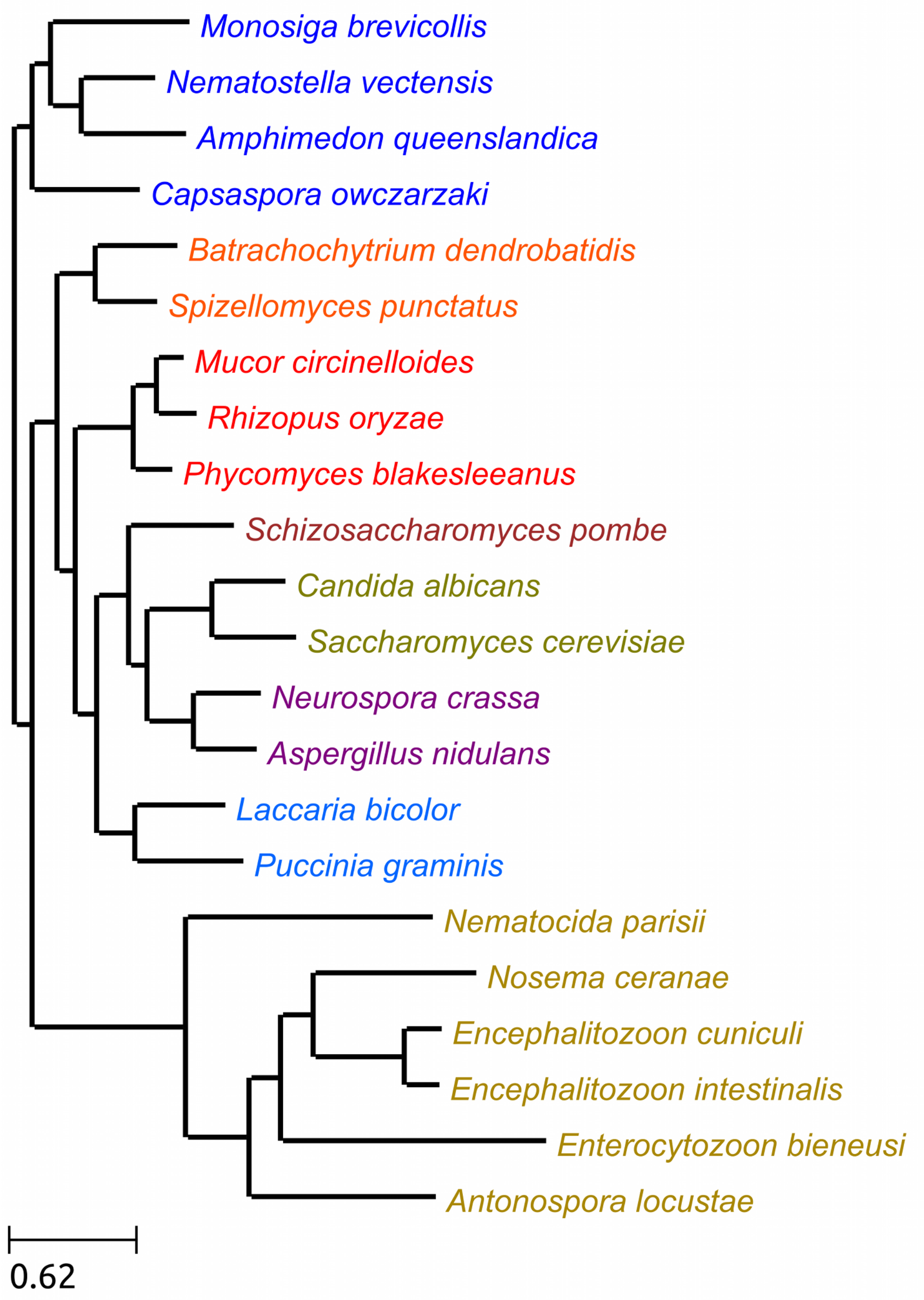
Monosiga brevicollis
Nematostella vectensis
Amphimedon queenslandica
Capsaspora owczarzaki
Batrachochytrium dendrobatidis
Spizellomyces punctatus
Mucor circinelloides
Rhizopus oryzae
Phycomyces blakesleeanus
Schizosaccharomyces pombe
Candida albicans
Saccharomyces cerevisiae
Neurospora crassa
Aspergillus nidulans
Laccaria bicolor
Puccinia graminis
Nematocida parisii
Nosema ceranae
Encephalitozoon cuniculi
Encephalitozoon intestinalis
Enterocytozoon bieneusi
Antonospora locustae
0.62

**Figure S13**

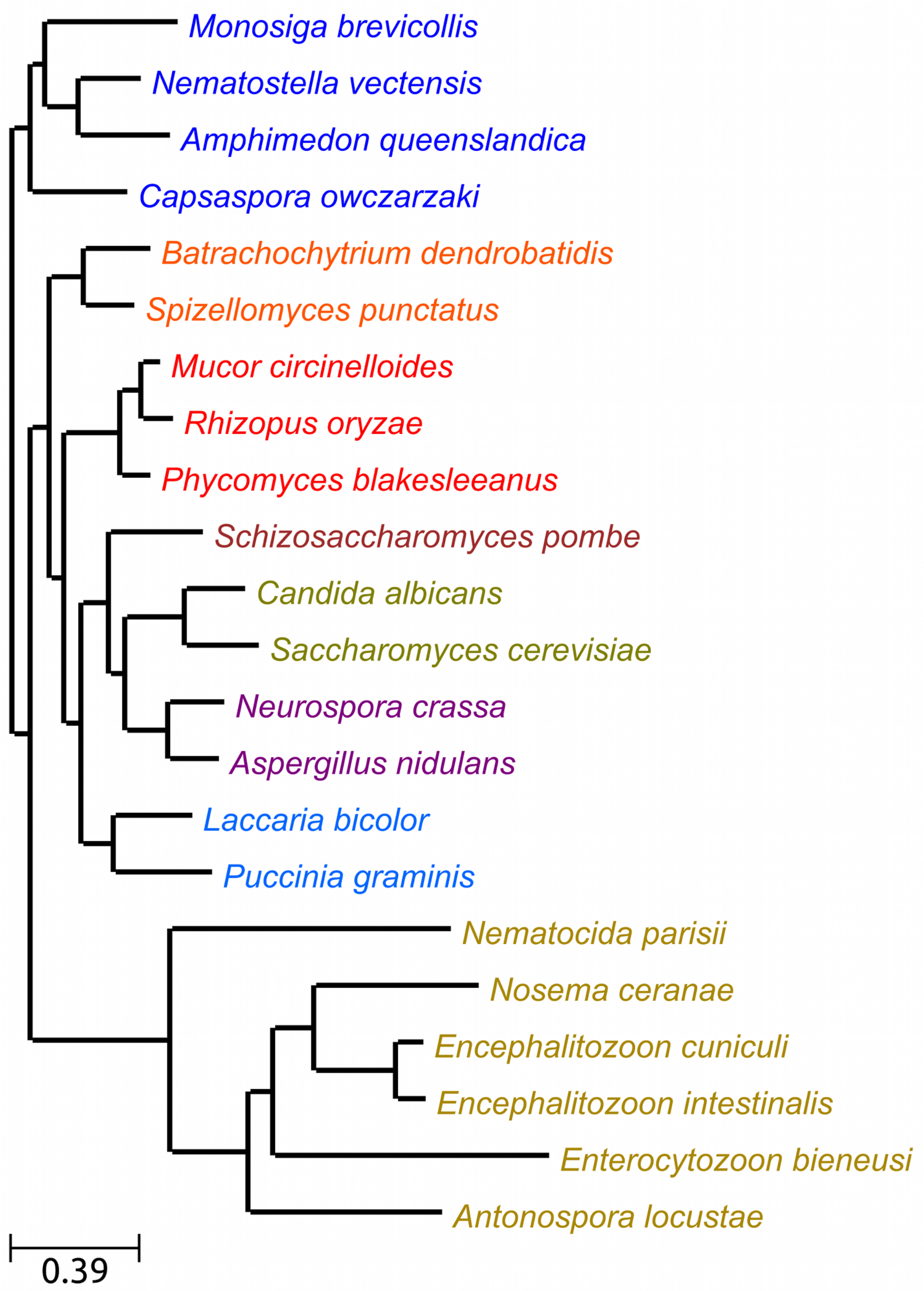
Monosiga brevicollis
Nematostella vectensis
Amphimedon queenslandica
Capsaspora owczarzaki
Batrachochytrium dendrobatidis
Spizellomyces punctatus
Mucor circinelloides
Rhizopus oryzae
Phycomyces blakesleeanus
Schizosaccharomyces pombe
Candida albicans
Saccharomyces cerevisiae
Neurospora crassa
Aspergillus nidulans
Laccaria bicolor
Puccinia graminis
Nematocida parisii
Nosema ceranae
Encephalitozoon cuniculi
Encephalitozoon intestinalis
Enterocytozoon bieneusi
Antonospora locustae
0.39

# Figure S14

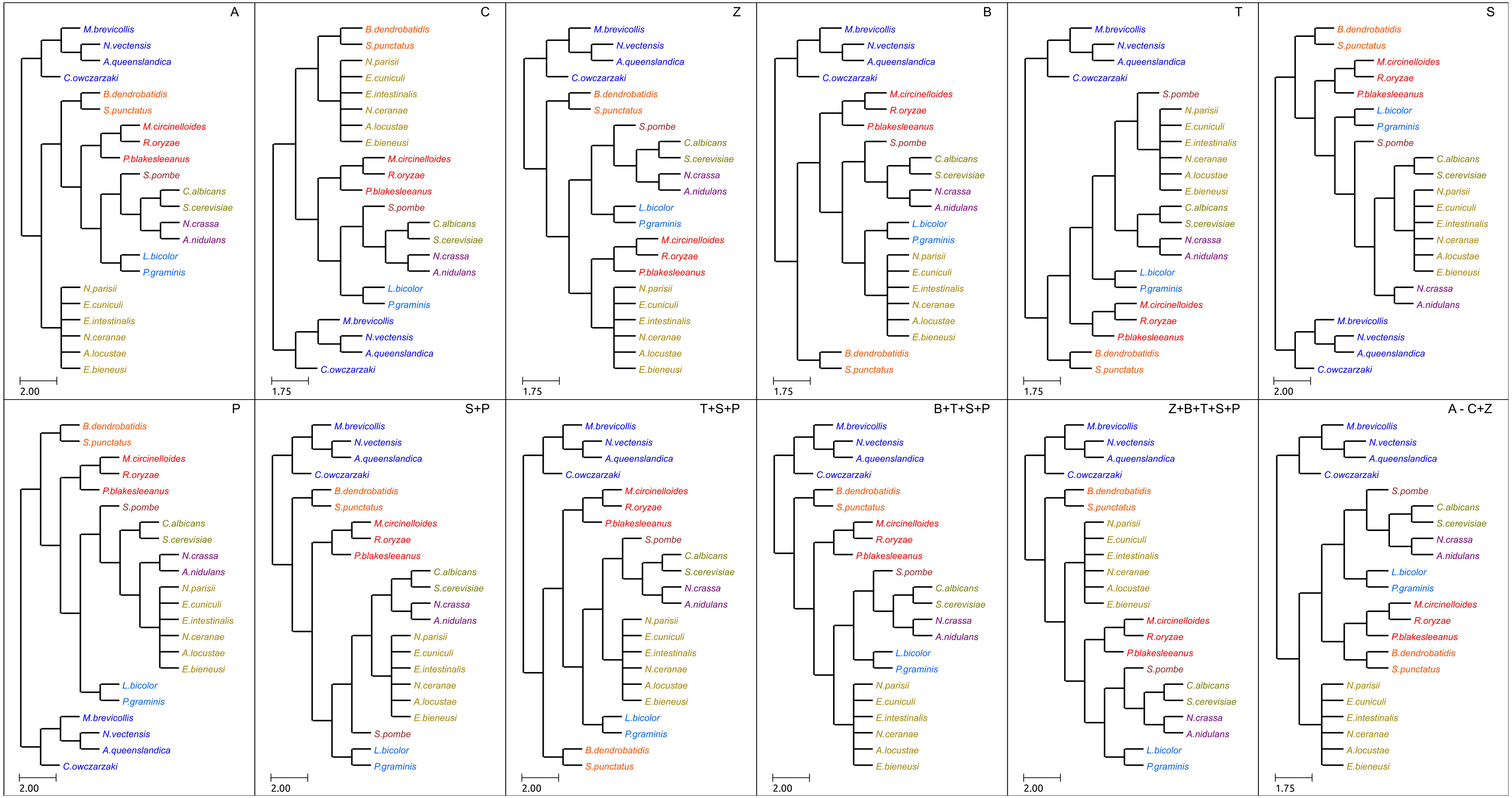
A
M.brevicollis
N.vectensis
A.queenslandica
C.owczarzaki
B.dendrobatidis
S.punctatus
M.circinelloides
R.oryzae
P.blakesleeanus
S.pombe
C.albicans
S.cerevisiae
N.crassa
A.nidulans
L.bicolor
P.graminis
N.parisii
E.cuniculi
E.intestinalis
N.ceranae
A.locustae
E.bieneusi
2.00
C
B.dendrobatidis
S.punctatus
N.parisii
E.cuniculi
E.intestinalis
N.ceranae
A.locustae
E.bieneusi
M.circinelloides
R.oryzae
P.blakesleeanus
S.pombe
C.albicans
S.cerevisiae
N.crassa
A.nidulans
L.bicolor
P.graminis
M.brevicollis
N.vectensis
A.queenslandica
C.owczarzaki
1.75
Z
M.brevicollis
N.vectensis
A.queenslandica
C.owczarzaki
B.dendrobatidis
S.punctatus
S.pombe
C.albicans
S.cerevisiae
N.crassa
A.nidulans
L.bicolor
P.graminis
M.circinelloides
R.oryzae
P.blakesleeanus
N.parisii
E.cuniculi
E.intestinalis
N.ceranae
A.locustae
E.bieneusi
1.75
B
M.brevicollis
N.vectensis
A.queenslandica
C.owczarzaki
M.circinelloides
R.oryzae
P.blakesleeanus
S.pombe
C.albicans
S.cerevisiae
N.crassa
A.nidulans
L.bicolor
P.graminis
N.parisii
E.cuniculi
E.intestinalis
N.ceranae
A.locustae
E.bieneusi
B.dendrobatidis
S.punctatus
1.75
T
M.brevicollis
N.vectensis
A.queenslandica
C.owczarzaki
S.pombe
N.parisii
E.cuniculi
E.intestinalis
N.ceranae
A.locustae
E.bieneusi
C.albicans
S.cerevisiae
N.crassa
A.nidulans
L.bicolor
P.graminis
M.circinelloides
R.oryzae
P.blakesleeanus
B.dendrobatidis
S.punctatus
1.75
S
B.dendrobatidis
S.punctatus
M.circinelloides
R.oryzae
P.blakesleeanus
L.bicolor
P.graminis
S.pombe
C.albicans
S.cerevisiae
N.parisii
E.cuniculi
E.intestinalis
N.ceranae
A.locustae
E.bieneusi
N.crassa
A.nidulans
M.brevicollis
N.vectensis
A.queenslandica
C.owczarzaki
2.00
P
B.dendrobatidis
S.punctatus
M.circinelloides
R.oryzae
P.blakesleeanus
S.pombe
C.albicans
S.cerevisiae
N.crassa
A.nidulans
N.parisii
E.cuniculi
E.intestinalis
N.ceranae
A.locustae
E.bieneusi
L.bicolor
P.graminis
M.brevicollis
N.vectensis
A.queenslandica
C.owczarzaki
2.00
S+P
M.brevicollis
N.vectensis
A.queenslandica
C.owczarzaki
B.dendrobatidis
S.punctatus
M.circinelloides
R.oryzae
P.blakesleeanus
C.albicans
S.cerevisiae
N.crassa
A.nidulans
N.parisii
E.cuniculi
E.intestinalis
N.ceranae
A.locustae
E.bieneusi
S.pombe
L.bicolor
P.graminis
2.00
T+S+P
M.brevicollis
N.vectensis
A.queenslandica
C.owczarzaki
M.circinelloides
R.oryzae
P.blakesleeanus
S.pombe
C.albicans
S.cerevisiae
N.crassa
A.nidulans
N.parisii
E.cuniculi
E.intestinalis
N.ceranae
A.locustae
E.bieneusi
L.bicolor
P.graminis
B.dendrobatidis
S.punctatus
2.00
B+T+S+P
M.brevicollis
N.vectensis
A.queenslandica
C.owczarzaki
B.dendrobatidis
S.punctatus
M.circinelloides
R.oryzae
P.blakesleeanus
S.pombe
C.albicans
S.cerevisiae
N.crassa
A.nidulans
L.bicolor
P.graminis
N.parisii
E.cuniculi
E.intestinalis
N.ceranae
A.locustae
E.bieneusi
2.00
Z+B+T+S+P
M.brevicollis
N.vectensis
A.queenslandica
C.owczarzaki
B.dendrobatidis
S.punctatus
N.parisii
E.cuniculi
E.intestinalis
N.ceranae
A.locustae
E.bieneusi
M.circinelloides
R.oryzae
P.blakesleeanus
S.pombe
C.albicans
S.cerevisiae
N.crassa
A.nidulans
L.bicolor
P.graminis
2.00
A - C+Z
M.brevicollis
N.vectensis
A.queenslandica
C.owczarzaki
S.pombe
C.albicans
S.cerevisiae
N.crassa
A.nidulans
L.bicolor
P.graminis
M.circinelloides
R.oryzae
P.blakesleeanus
B.dendrobatidis
S.punctatus
N.parisii
E.cuniculi
E.intestinalis
N.ceranae
A.locustae
E.bieneusi
1.75

## Figure S15

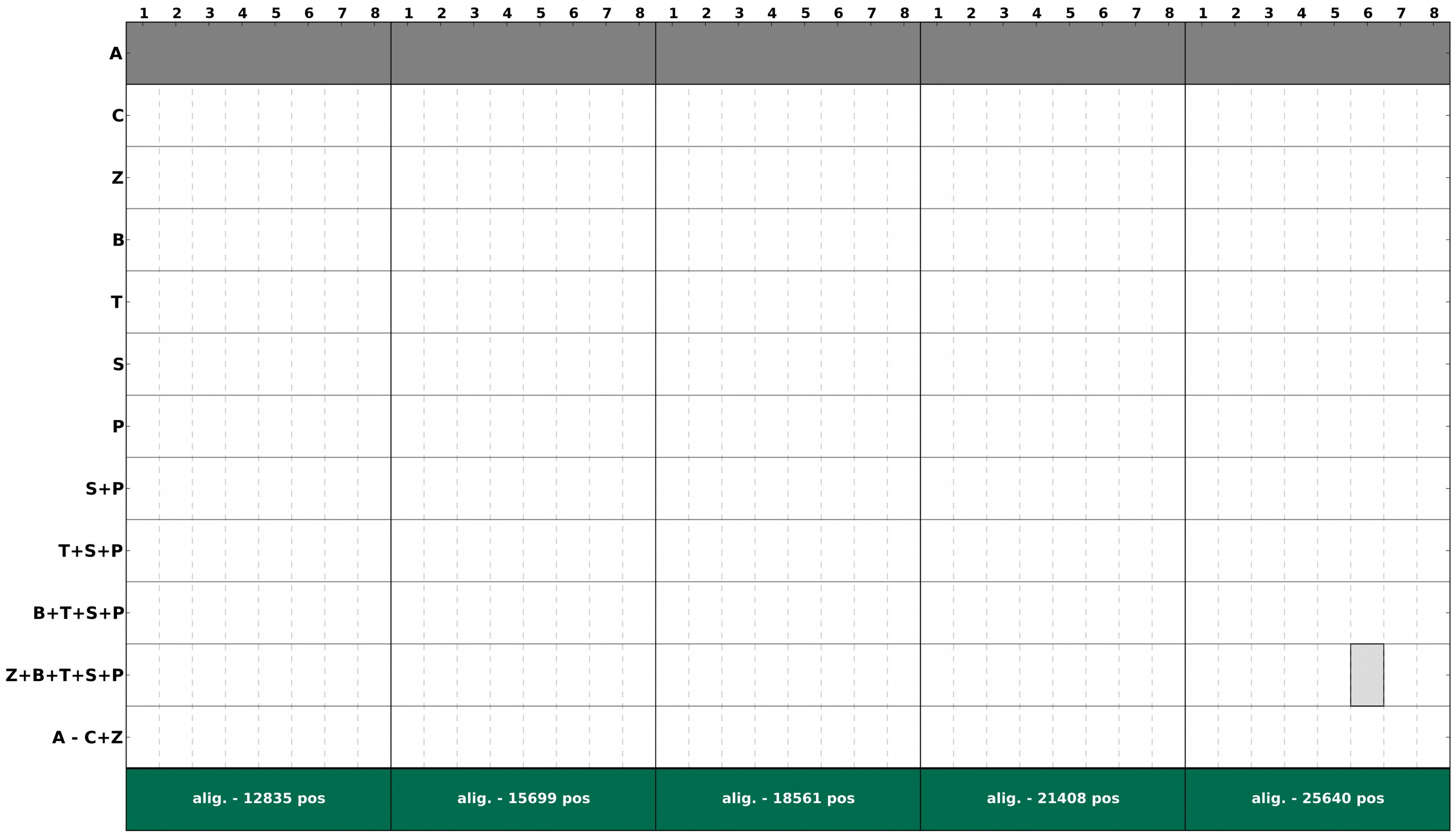
1 2 3 4 5 6 7 8
A
C
Z
B
T
S
P
S+P
T+S+P
B+T+S+P
Z+B+T+S+P
A - C+Z
alig. - 12835 pos
alig. - 15699 pos
alig. - 18561 pos
alig. - 21408 pos
alig. - 25640 pos

# Figure S16

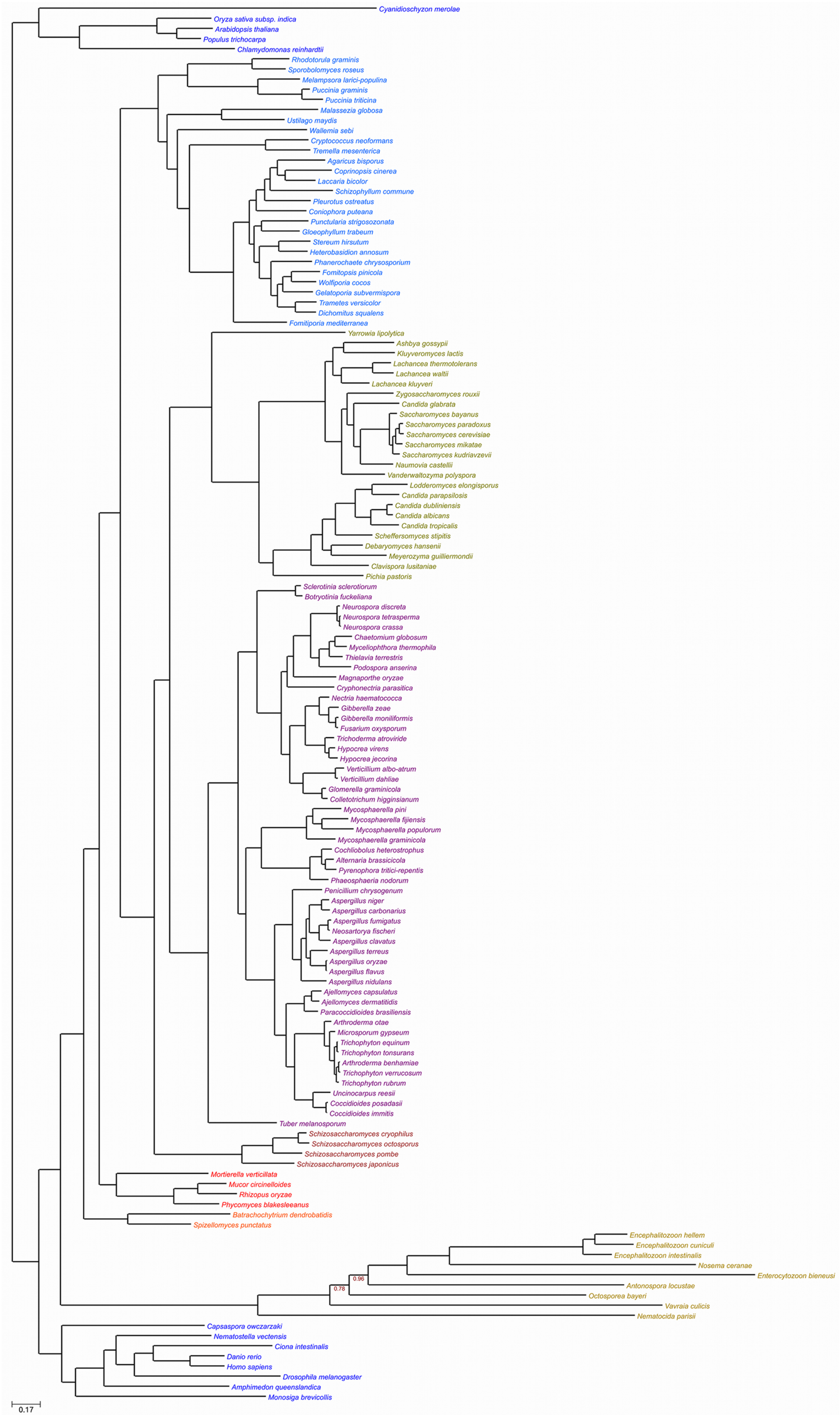

Cyanidioschyzon merolae
Oryza sativa subsp. indica
Arabidopsis thaliana
Populus trichocarpa
Chlamydomonas reinhardtii
Rhodotorula graminis
Sporobolomyces roseus
Melampsora larici-populina
Puccinia graminis
Puccinia triticina
Malassezia globosa
Ustilago maydis
Wallemia sebi
Cryptococcus neoformans
Tremella mesenterica
Agaricus bisporus
Coprinopsis cinerea
Laccaria bicolor
Schizophyllum commune
Pleurotus ostreatus
Coniophora puteana
Punctularia strigosozonata
Gloeophyllum trabeum
Stereum hirsutum
Heterobasidion annosum
Phanerochaete chrysosporium
Fomitopsis pinicola
Wolfiporia cocos
Gelatoporia subvermispora
Trametes versicolor
Dichomitus squalens
Fomitiporia mediterranea
Yarrowia lipolytica
Ashbya gossypii
Kluyveromyces lactis
Lachancea thermotolerans
Lachancea waltii
Lachancea kluyveri
Zygosaccharomyces rouxii
Candida glabrata
Saccharomyces bayanus
Saccharomyces paradoxus
Saccharomyces cerevisiae
Saccharomyces mikatae
Saccharomyces kudriavzevii
Naumovia castellii
Vanderwaltozyma polyspora
Lodderomyces elongisporus
Candida parapsilosis
Candida dubliniensis
Candida albicans
Candida tropicalis
Scheffersomyces stipitis
Debaryomyces hansenii
Meyerozyma guilliermondii
Clavispora lusitaniae
Pichia pastoris
Sclerotinia sclerotiorum
Botryotinia fuckeliana
Neurospora discreta
Neurospora tetrasperma
Neurospora crassa
Chaetomium globosum
Myceliophthora thermophila
Thielavia terrestris
Podospora anserina
Magnaporthe oryzae
Cryphonectria parasitica
Nectria haematococca
Gibberella zeae
Gibberella moniliformis
Fusarium oxysporum
Trichoderma atroviride
Hypocrea virens
Hypocrea jecorina
Verticillium albo-atrum
Verticillium dahliae
Glomerella graminicola
Colletotrichum higginsianum
Mycosphaerella pini
Mycosphaerella fijiensis
Mycosphaerella populorum
Mycosphaerella graminicola
Cochliobolus heterostrophus
Alternaria brassicicola
Pyrenophora tritici-repentis
Phaeosphaeria nodorum
Penicillium chrysogenum
Aspergillus niger
Aspergillus carbonarius
Aspergillus fumigatus
Neosartorya fischeri
Aspergillus clavatus
Aspergillus terreus
Aspergillus oryzae
Aspergillus flavus
Aspergillus nidulans
Ajellomyces capsulatus
Ajellomyces dermatitidis
Paracoccidioides brasiliensis
Arthroderma otae
Microsporum gypseum
Trichophyton equinum
Trichophyton tonsurans
Arthroderma benhamiae
Trichophyton verrucosum
Trichophyton rubrum
Uncinocarpus reesii
Coccidioides posadasii
Coccidioides immitis
Tuber melanosporum
Schizosaccharomyces cryophilus
Schizosaccharomyces octosporus
Schizosaccharomyces pombe
Schizosaccharomyces japonicus
Mortierella verticillata
Mucor circinelloides
Rhizopus oryzae
Phycomyces blakesleeanus
Batrachochytrium dendrobatidis
Spizellomyces punctatus
Encephalitozoon hellem
Encephalitozoon cuniculi
Encephalitozoon intestinalis
Nosema ceranae
Enterocytozoon bieneusi
0.96
0.78
Antonospora locustae
Octosporea bayeri
Vavraia culicis
Nematocida parisii
Capsaspora owczarzaki
Nematostella vectensis
Ciona intestinalis
Danio rerio
Homo sapiens
Drosophila melanogaster
Amphimedon queenslandica
Monosiga brevicollis
0.17

## Figure S17

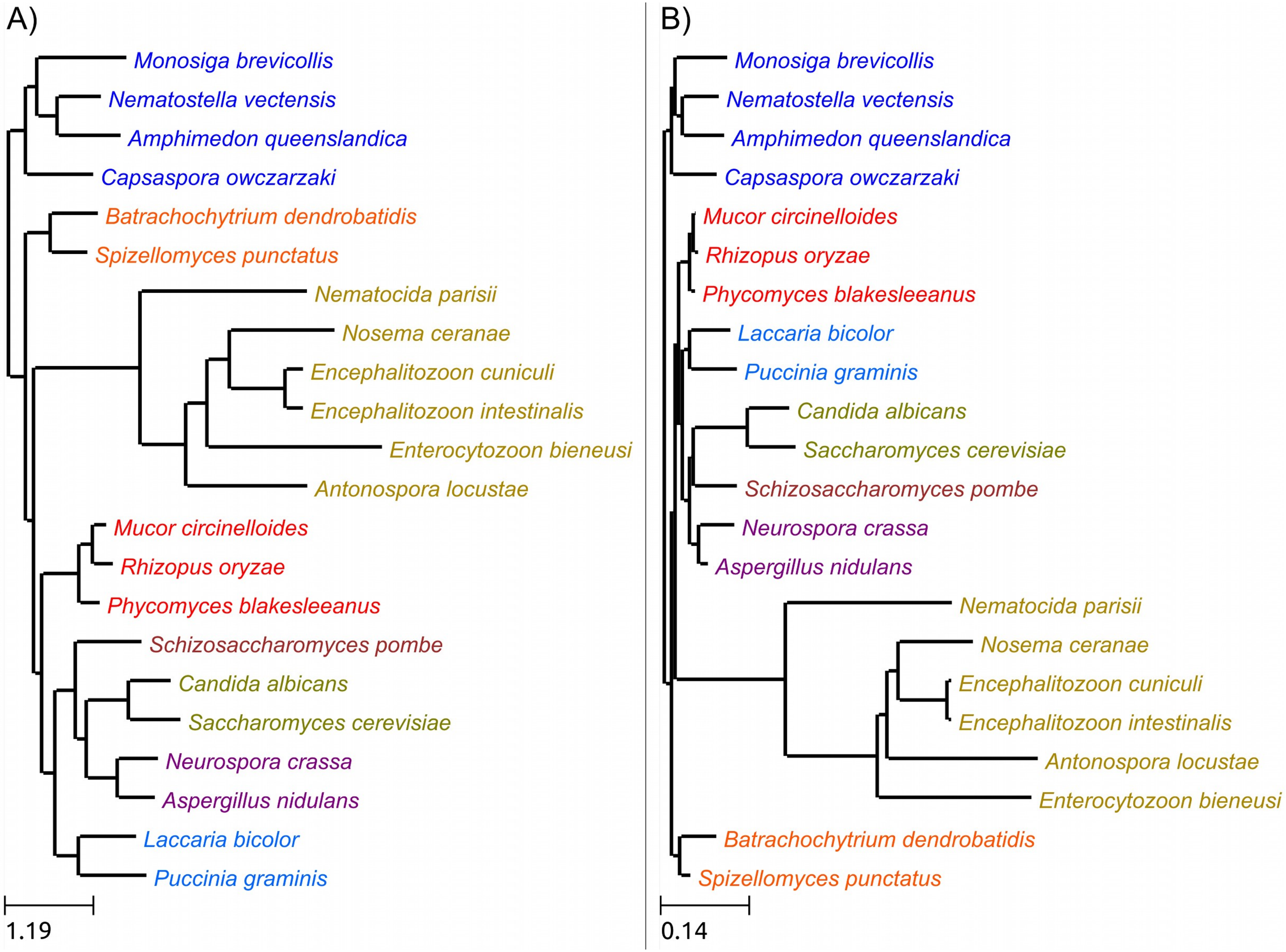

A)
Monosiga brevicollis
Nematostella vectensis
Amphimedon queenslandica
Capsaspora owczarzaki
Batrachochytrium dendrobatidis
Spizellomyces punctatus
Nematocida parisii
Nosema ceranae
Encephalitozoon cuniculi
Encephalitozoon intestinalis
Enterocytozoon bieneusi
Antonospora locustae
Mucor circinelloides
Rhizopus oryzae
Phycomyces blakesleeanus
Schizosaccharomyces pombe
Candida albicans
Saccharomyces cerevisiae
Neurospora crassa
Aspergillus nidulans
Laccaria bicolor
Puccinia graminis
1.19
B)
Monosiga brevicollis
Nematostella vectensis
Amphimedon queenslandica
Capsaspora owczarzaki
Mucor circinelloides
Rhizopus oryzae
Phycomyces blakesleeanus
Laccaria bicolor
Puccinia graminis
Candida albicans
Saccharomyces cerevisiae
Schizosaccharomyces pombe
Neurospora crassa
Aspergillus nidulans
Nematocida parisii
Nosema ceranae
Encephalitozoon cuniculi
Encephalitozoon intestinalis
Antonospora locustae
Enterocytozoon bieneusi
Batrachochytrium dendrobatidis
Spizellomyces punctatus
0.14

# Table S1. Relaxed Synteny

Intervening genes: both genomes. Windows's size reference genome: +3 Window's size compared genome: +4. Surronding homologs: 15 genes

| | | *Antonospora locustae* (Alo) | | | | | *Enterocytozoon bieneusi* (Ebi) | | | | | *Encephalitozoon cuniculi* (Ecu) | | | | |
|---|---|---|---|---|---|---|---|---|---|---|---|---|---|---|---|---|
| | | 1 | 2 | 3 | 4 | 5 | 1 | 2 | 3 | 4 | 5 | 1 | 2 | 3 | 4 | 5 |
| **Microsporidia** | **Alo** | -- | -- | **--** | -- | **--** | 745 | 131 | **175.84** | 127 | **170.47** | 1113 | 340 | **305.48** | 321 | **288.41** |
| | **Ebi** | 879 | 77 | **87.60** | 68 | **77.36** | -- | -- | **--** | -- | **--** | 1609 | 673 | **418.27** | 616 | **382.85** |
| | **Ecu** | 851 | 283 | **332.55** | 260 | **305.52** | 987 | 626 | **634.25** | 600 | **607.90** | -- | -- | **--** | -- | **--** |
| | **Eit** | 780 | 166 | **212.82** | 152 | **194.87** | 903 | 576 | **637.87** | 549 | **607.97** | 1612 | 6540 | **4057.1** | 6234 | **3867.2** |
| | **Nce** | 722 | 91 | **126.04** | 88 | **121.88** | 850 | 344 | **404.71** | 323 | **380.00** | 1308 | 1129 | **863.15** | 1099 | **840.21** |
| | **Npa** | 644 | 65 | **100.93** | 65 | **100.93** | 718 | 131 | **182.45** | 131 | **182.45** | 945 | 271 | **286.77** | 265 | **280.42** |
| **Chytridiomycota** | **Bde** | 1228 | 17 | **13.84** | 10 | **8.14** | 1199 | 36 | **30.03** | 24 | **20.02** | 1593 | 46 | **28.88** | 36 | **22.60** |
| | **Spc** | 1434 | 20 | **13.95** | 18 | **12.55** | 1377 | 19 | **13.80** | 16 | **11.62** | 1774 | 44 | **24.80** | 34 | **19.17** |
| **Zygomycotina** | **Mci** | 1910 | 46 | **24.08** | 24 | **12.57** | 1897 | 44 | **23.19** | 23 | **12.12** | 2481 | 63 | **25.39** | 45 | **18.14** |
| | **Pbl** | 1760 | 41 | **23.30** | 24 | **13.64** | 1763 | 56 | **31.76** | 21 | **11.91** | 2272 | 55 | **24.21** | 40 | **17.61** |
| | **Ror** | 2011 | 35 | **17.40** | 19 | **9.45** | 2069 | 41 | **19.82** | 26 | **12.57** | 2636 | 61 | **23.14** | 40 | **15.17** |
| **Basidiomycotina** | **Lbi** | 1355 | 13 | **9.59** | 11 | **8.12** | 1338 | 11 | **8.22** | 10 | **7.47** | 1764 | 56 | **31.75** | 23 | **13.04** |
| | **Pgr** | 1050 | 12 | **11.43** | 4 | **3.81** | 1072 | 7 | **6.53** | 7 | **6.53** | 1399 | 14 | **10.01** | 13 | **9.29** |
| **Taphrinomycotina** | **Spb** | 1037 | 20 | **19.29** | 14 | **13.50** | 1064 | 24 | **22.56** | 20 | **18.80** | 1376 | 41 | **29.80** | 33 | **23.98** |
| **Pezizomycotina** | **Ani** | 1087 | 13 | **11.96** | 13 | **11.96** | 1059 | 14 | **13.22** | 13 | **12.28** | 1348 | 28 | **20.77** | 23 | **17.06** |
| | **Ncr** | 1070 | 13 | **12.15** | 8 | **7.48** | 1051 | 7 | **6.66** | 7 | **6.66** | 1352 | 25 | **18.49** | 20 | **14.79** |
| **Saccharomycotina** | **Cal** | 920 | 14 | **15.22** | 12 | **13.04** | 961 | 18 | **18.73** | 17 | **17.69** | 1205 | 31 | **25.73** | 28 | **23.24** |
| | **Sce** | 1026 | 26 | **25.34** | 12 | **11.70** | 1082 | 28 | **25.88** | 19 | **17.56** | 1362 | 48 | **35.24** | 35 | **25.70** |

1) Number of homologous genes between both species.

2) Sintenic pairs applied the relaxed method.

3) Corrected sintenic pairs by homologous number/1000.

4) Sintenic pairs discarding paralogous pairs.

5) Corrected sintenic pairs by homologous number/1000 and discarding paralogous pairs.

| | | *Encephalitozoon intestinalis* (Eit) | | | | | *Nosema ceranae* (Nce) | | | | | *Nematocida parisii* (Npa) | | | | |
|---|---|---|---|---|---|---|---|---|---|---|---|---|---|---|---|---|
| | | 1 | 2 | 3 | 4 | 5 | 1 | 2 | 3 | 4 | 5 | 1 | 2 | 3 | 4 | 5 |
| **Microsporidia** | **Alo** | 706 | 119 | **168.56** | 114 | **161.47** | 703 | 85 | **120.91** | 81 | **115.22** | 798 | 89 | **111.53** | 87 | **109.02** |
| | **Ebi** | 999 | 334 | **334.33** | 306 | **306.31** | 1112 | 240 | **215.83** | 221 | **198.74** | 1171 | 148 | **126.39** | 130 | **111.02** |
| | **Ecu** | 1038 | 6182 | **5955.7** | 5890 | **5674.4** | 949 | 904 | **952.58** | 874 | **920.97** | 976 | 271 | **277.66** | 262 | **268.44** |
| | **Eit** | -- | -- | **--** | -- | **--** | 867 | 581 | **670.13** | 564 | **650.52** | 903 | 262 | **290.14** | 255 | **282.39** |
| | **Nce** | 790 | 449 | **568.35** | 431 | **545.57** | -- | -- | **--** | -- | **--** | 843 | 133 | **157.77** | 130 | **154.21** |
| | **Npa** | 659 | 138 | **209.41** | 134 | **203.34** | 660 | 64 | **96.97** | 64 | **96.97** | -- | -- | **--** | -- | **--** |
| **Chytridiomycota** | **Bde** | 1228 | 39 | **31.76** | 26 | **21.17** | 1214 | 37 | **30.48** | 23 | **18.95** | 1476 | 32 | **21.68** | 30 | **20.33** |
| | **Spc** | 1434 | 27 | **18.83** | 24 | **16.74** | 1443 | 25 | **17.33** | 17 | **11.78** | 1681 | 29 | **17.25** | 26 | **15.47** |
| **Zygomycotina** | **Mci** | 1988 | 54 | **27.16** | 37 | **18.61** | 1933 | 57 | **29.49** | 32 | **16.55** | 2334 | 33 | **14.14** | 20 | **8.57** |
| | **Pbl** | 1834 | 48 | **26.17** | 32 | **17.45** | 1831 | 69 | **37.68** | 27 | **14.75** | 2177 | 33 | **15.16** | 24 | **11.02** |
| | **Ror** | 2119 | 53 | **25.01** | 38 | **17.93** | 3142 | 70 | **22.28** | 31 | **9.87** | 2516 | 38 | **15.10** | 22 | **8.74** |
| **Basidiomycotina** | **Lbi** | 1400 | 43 | **30.71** | 12 | **8.57** | 1405 | 18 | **12.81** | 15 | **10.68** | 1655 | 23 | **13.90** | 19 | **11.48** |
| | **Pgr** | 1046 | 6 | **5.74** | 6 | **5.74** | 1148 | 5 | **4.36** | 4 | **3.48** | 1299 | 14 | **10.78** | 7 | **5.39** |
| **Taphrinomycotina** | **Spb** | 1058 | 34 | **32.14** | 24 | **22.68** | 1048 | 29 | **27.67** | 22 | **20.99** | 1335 | 33 | **24.72** | 26 | **19.48** |
| **Pezizomycotina** | **Ani** | 1055 | 18 | **17.06** | 17 | **16.11** | 1055 | 15 | **14.22** | 14 | **13.27** | 1281 | 29 | **22.64** | 26 | **20.30** |
| | **Ncr** | 1070 | 20 | **18.69** | 18 | **16.82** | 1029 | 14 | **13.61** | 12 | **11.66** | 1310 | 26 | **19.85** | 20 | **15.27** |
| **Saccharomycotina** | **Cal** | 924 | 22 | **23.81** | 19 | **20.56** | 920 | 21 | **22.83** | 14 | **15.22** | 1186 | 20 | **16.86** | 18 | **15.18** |
| | **Sce** | 1042 | 30 | **28.79** | 22 | **21.11** | 1061 | 33 | **31.10** | 23 | **21.68** | 1345 | 39 | **29.00** | 29 | **21.56** |

1) Number of homologous genes between both species.

2) Sintenic pairs applied the relaxed method.

3) Corrected sintenic pairs by homologous number/1000.

4) Sintenic pairs discarding paralogous pairs.

5) Corrected sintenic pairs by homologous number/1000 and discarding paralogous pairs.

# Table S2. Strict Sinteny

Intervening genes: both genomes. Windows size: +3

| | | *Antonospora locustae* (Alo) | | | | | *Enterocytozoon bieneusi* (Ebi) | | | | | *Encephalitozoon cuniculi* (Ecu) | | | | |
|---|---|---|---|---|---|---|---|---|---|---|---|---|---|---|---|---|
| | | 1 | 2 | 3 | 4 | 5 | 1 | 2 | 3 | 4 | 5 | 1 | 2 | 3 | 4 | 5 |
| **Microsporidia** | **Alo** | -- | -- | **--** | -- | **--** | 551 | 52 | **94.37** | 52 | **94.37** | 919 | 182 | **198.04** | 178 | **193.69** |
| | **Ebi** | 682 | 60 | **87.98** | 56 | **82.11** | -- | -- | **--** | -- | **--** | 1160 | 340 | **293.10** | 334 | **287.93** |
| | **Ecu** | 845 | 183 | **216.57** | 177 | **209.47** | 810 | 373 | **460.49** | 372 | **459.26** | -- | -- | **--** | -- | **--** |
| | **Eit** | 771 | 163 | **211.41** | 158 | **204.93** | 752 | 347 | **461.44** | 347 | **461.44** | 1344 | 4386 | **3263.4** | 4352 | **3238.1** |
| | **Nce** | 667 | 58 | **86.96** | 58 | **86.96** | 691 | 119 | **172.21** | 118 | **170.77** | 1159 | 532 | **459.02** | 531 | **458.15** |
| | **Npa** | 534 | 52 | **97.38** | 52 | **97.38** | 546 | 89 | **163.00** | 89 | **163.00** | 771 | 156 | **202.33** | 156 | **202.33** |
| **Chytridiomycota** | **Bde** | 510 | 5 | **9.80** | 5 | **9.80** | 449 | 8 | **17.82** | 8 | **17.82** | 650 | 6 | **9.23** | 6 | **9.23** |
| | **Spc** | 566 | 4 | **7.07** | 4 | **7.07** | 497 | 5 | **10.06** | 5 | **10.06** | 722 | 5 | **6.93** | 5 | **6.93** |
| **Zygomycotina** | **Mci** | 760 | 5 | **6.58** | 4 | **5.26** | 679 | 17 | **25.04** | 11 | **16.20** | 1005 | 16 | **15.92** | 15 | **14.93** |
| | **Pbl** | 724 | 4 | **5.52** | 4 | **5.52** | 636 | 5 | **7.86** | 4 | **6.29** | 938 | 8 | **8.53** | 7 | **7.46** |
| | **Ror** | 787 | 4 | **5.08** | 2 | **2.54** | 751 | 6 | **7.99** | 6 | **7.99** | 1042 | 11 | **10.56** | 9 | **8.64** |
| **Basidiomycotina** | **Lbi** | 542 | 4 | **7.38** | 2 | **3.69** | 470 | 6 | **12.77** | 6 | **12.77** | 713 | 10 | **14.03** | 8 | **11.22** |
| | **Pgr** | 416 | 2 | **4.81** | 2 | **4.81** | 357 | 4 | **11.20** | 4 | **11.20** | 533 | 4 | **7.50** | 4 | **7.50** |
| **Taphrinomycotina** | **Spb** | 502 | 4 | **7.97** | 4 | **7.97** | 477 | 7 | **14.68** | 7 | **14.68** | 659 | 8 | **12.14** | 8 | **12.14** |
| **Pezizomycotina** | **Ani** | 442 | 3 | **6.79** | 3 | **6.79** | 394 | 6 | **15.23** | 6 | **15.23** | 573 | 6 | **10.47** | 6 | **10.47** |
| | **Ncr** | 457 | 1 | **2.19** | 1 | **2.19** | 409 | 8 | **19.56** | 8 | **19.56** | 606 | 6 | **9.90** | 6 | **9.90** |
| **Saccharomycotina** | **Cal** | 419 | -- | **--** | -- | **--** | 422 | 8 | **18.96** | 8 | **18.96** | 566 | 10 | **17.67** | 10 | **17.67** |
| | **Sce** | 477 | 4 | **8.39** | 4 | **8.39** | 467 | 7 | **14.99** | 6 | **12.85** | 627 | 6 | **9.57** | 5 | **7.97** |

1) Number of orthologous genes between both species.

2) Sintenic pairs applied the strict method.

3) Corrected sintenic pairs by orthologous number/1000.

4) Sintenic pairs discarding paralogous pairs.

5) Corrected sintenic pairs by orthologous number/1000 and discarding paralogous pairs.

| | | *Encephalitozoon intestinalis* (Eit) | | | | | *Nosema ceranae* (Nce) | | | | | *Nematocida parisii* (Npa) | | | | |
|---|---|---|---|---|---|---|---|---|---|---|---|---|---|---|---|---|
| | | 1 | 2 | 3 | 4 | 5 | 1 | 2 | 3 | 4 | 5 | 1 | 2 | 3 | 4 | 5 |
| **Microsporidia** | **Alo** | 852 | 158 | **185.45** | 155 | **181.92** | 732 | 62 | **84.70** | 60 | **81.97** | 570 | 31 | **54.39** | 31 | **54.39** |
| | **Ebi** | 1086 | 319 | **293.74** | 312 | **287.29** | 1056 | 136 | **128.79** | 133 | **125.95** | 760 | 57 | **75.00** | 56 | **73.68** |
| | **Ecu** | 1424 | 4301 | **3020.4** | 4241 | **2978.2** | 1167 | 563 | **482.43** | 563 | **482.43** | 787 | 126 | **160.10** | 126 | **160.10** |
| | **Eit** | -- | -- | **--** | -- | **--** | 1083 | 539 | **497.69** | 539 | **497.69** | 733 | 132 | **180.08** | 132 | **180.08** |
| | **Nce** | 1087 | 515 | **473.78** | 512 | **471.02** | -- | -- | **--** | -- | **--** | 654 | 46 | **70.34** | 46 | **70.34** |
| | **Npa** | 724 | 161 | **222.38** | 161 | **222.38** | 671 | 67 | **99.85** | 67 | **99.85** | -- | -- | **--** | -- | **--** |
| **Chytridiomycota** | **Bde** | 609 | 10 | **16.42** | 10 | **16.42** | 567 | 9 | **15.87** | 9 | **15.87** | 582 | 10 | **17.18** | 10 | **17.18** |
| | **Spc** | 696 | 10 | **14.37** | 10 | **14.37** | 612 | 3 | **4.90** | 3 | **4.90** | 635 | 5 | **7.87** | 5 | **7.87** |
| **Zygomycotina** | **Mci** | 965 | 18 | **18.65** | 17 | **17.62** | 869 | 7 | **8.06** | 6 | **6.90** | 881 | 8 | **9.08** | 8 | **9.08** |
| | **Pbl** | 900 | 8 | **8.89** | 8 | **8.89** | 832 | 3 | **3.61** | 3 | **3.61** | 846 | 6 | **7.09** | 6 | **7.09** |
| | **Ror** | 1026 | 16 | **15.59** | 14 | **13.65** | 1564 | 9 | **5.75** | 6 | **3.84** | 943 | 9 | **9.54** | 8 | **8.48** |
| **Basidiomycotina** | **Lbi** | 676 | 9 | **13.31** | 8 | **11.83** | 599 | 7 | **11.69** | 5 | **8.35** | 622 | 2 | **3.22** | 2 | **3.22** |
| | **Pgr** | 523 | 5 | **9.56** | 5 | **9.56** | 522 | 6 | **11.49** | 6 | **11.49** | 488 | 4 | **8.20** | 3 | **6.15** |
| **Taphrinomycotina** | **Spb** | 644 | 9 | **13.98** | 9 | **13.98** | 590 | 3 | **5.08** | 3 | **5.08** | 622 | 4 | **6.43** | 4 | **6.43** |
| **Pezizomycotina** | **Ani** | 556 | 6 | **10.79** | 6 | **10.79** | 497 | 2 | **4.02** | 2 | **4.02** | 519 | 3 | **5.78** | 3 | **5.78** |
| | **Ncr** | 568 | 5 | **8.80** | 5 | **8.80** | 505 | 3 | **5.94** | 3 | **5.94** | 539 | 3 | **5.57** | 3 | **5.57** |
| **Saccharomycotina** | **Cal** | 534 | 9 | **16.85** | 9 | **16.85** | 487 | 11 | **22.59** | 6 | **12.32** | 522 | 6 | **11.49** | 6 | **11.49** |
| | **Sce** | 593 | 9 | **15.18** | 8 | **13.49** | 558 | 6 | **10.75** | 5 | **8.96** | 592 | 5 | **8.45** | 4 | **6.76** |

1) Number of orthologous genes between both species.

2) Sintenic pairs applied the strict method.

3) Corrected sintenic pairs by orthologous number/1000.

4) Sintenic pairs discarding paralogous pairs.

5) Corrected sintenic pairs by orthologous number/1000 and discarding paralogous pairs.

# Table S3. Analyses performed

| Analyses | | | | | Main conclusion |
|---|---|---|---|---|---|
| **A.- Chromosomal neighborhood conservation** | A1.- Strict synteny | | | | Similar low levels of gene order conservation between Microsporidia and other fungal groups |
| | A2.- Relaxed synteny | | | | |
| **B.- Phylome analysis** | B1.- Super tree | | | | Early branching position of Microsporidia |
| | B2.-Sister group analysis | Tree filtering | Monophyletic out-groups | | The early branching position of Microsporidia is the best supported hypothesis. The support increases with the filters |
| | | | Alignment quality | | |
| | | | Branch support | | |
| | | | Alignment length | | |
| | | | Combination of all filters | | |
| **C.- Gene concatenation analysis** ***(Concatenation of 53 wide-spread, single copy, genes)*** | C1.- Whole alignment | | Tree reconstruction: Maximum Likelihood (CAT, LG and Covarion models) Bayesian (CAT model) Sequence re-coding to reduced alphabet + Maximum likelihood (GTR model) | Comparison of alternative topologies (CONSEL) | Early branching position of Microsporidia The support of alternative topologies is always significantly lower |
| | C2.- C1 + Remove compositional bias (BMGE) | | | | |
| | C3.- Remove non-informative sites in microsporidia | | | | |
| | C4.- C3 + Remove compositional bias (BMGE) | | | | |
| | Partitioning of alignments C1, C2, C3 and C4 | C5.- Sequential removal of fast evolving positions (4 sub-alignments) | Tree reconstruction: Maximum likelihood (LG model) | | |
| | | C6.- Division by divergence in microsporidia (4 sub-alignments) | | | |
| | Extended phylogenies | C7 .- Addition of 3 microsporidian species | | | |
| | | C8 .- C7 + 113 species | | | |
| **D.- Additional Long Branch Attraction tests on C3** | D1.- Replace microsporidia by random sequences | | | | Results are likely not due to long branch attraction |
| | D2.- Simulations | | | | |

# Table S4. Proteomes Information

| Taxonomic Group | Code | Scientific Species Name | Source | As of |
|---|---|---|---|---|
| Microsporidia | Alo | Antonospora locustae | A.locustae DB | 06/2011 |
| Microsporidia | Ecu | Encephalitozoon cuniculi | Broad Institute | 06/2011 |
| Microsporidia | Ebi | Enterocytozoon bieneusi | MicrosporidiaDB | 06/2011 |
| Microsporidia | Nce | Nosema ceranae | Broad Institute | 06/2011 |
| Microsporidia | Eit | Encephalitozoon intestinalis | Broad Institute | 06/2011 |
| Microsporidia | Npa | Nematocida parisii | Broad Institute | 06/2011 |
| Chytridiomycota | Spc | Spizellomyces punctatus | Broad Institute | 06/2011 |
| Chytridiomycota | Bde | Batrachochytrium dendrobatidis | JGI | 06/2011 |
| Zygomycota | Pbl | Phycomyces blakesleeanus | JGI | 06/2011 |
| Zygomycota | Mci | Mucor circinelloides | JGI | 06/2011 |
| Zygomycota | Ror | Rhizopus oryzae | Broad Institute | 06/2011 |
| Basidiomycota | Lbi | Laccaria bicolor | JGI | 06/2011 |
| Basidiomycota | Pgr | Puccinia graminis | JGI | 06/2011 |
| Schizosaccharomycetes | Spb | Schizosaccharomyces pombe | Broad Institute | 06/2011 |
| Saccharomycotina | Cal | Candida albicans | Broad Institute | 06/2011 |
| Saccharomycotina | Sce | Saccharomyces cerevisiae | SGD | 06/2011 |
| Pezizomycotina | Ncr | Neurospora crassa | JGI | 06/2011 |
| Pezizomycotina | Ani | Aspergillus nidulans | JGI | 06/2011 |
| Metazoa; Eumetazoa | Nem | Nematostella vectensis | JGI | 06/2011 |
| Metazoa; Porifera | Aqu | Amphimedon queenslandica | JGI | 06/2011 |
| Choanoflagellida | Mbr | Monosiga brevicollis | JGI | 06/2011 |
| Capsaspora | Cwc | Capsaspora owczarzaki | Broad Institute | 06/2011 |
| Basidiomycota | Cci | Coprinopsis cinerea | JGI | 06/2011 |
| Basidiomycota | Abi | Agaricus bisporus | JGI | 06/2011 |
| Basidiomycota | Scm | Schizophyllum commune | JGI | 06/2011 |
| Basidiomycota | Mpr | Moniliophthora perniciosa | JGI | 06/2011 |
| Basidiomycota | Pos | Pleurotus ostreatus | JGI | 06/2011 |
| Basidiomycota | Cpt | Coniophora puteana | JGI | 06/2011 |
| Basidiomycota | Han | Heterobasidion annosum | JGI | 06/2011 |
| Basidiomycota | Shi | Stereum hirsutum | JGI | 06/2011 |
| Basidiomycota | Gtr | Gloeophyllum trabeum | JGI | 06/2011 |
| Basidiomycota | Psa | Punctularia strigosozonata | JGI | 06/2011 |
| Basidiomycota | Pch | Phanerochaete chrysosporium | JGI | 06/2011 |
| Basidiomycota | Tvr | Trametes versicolor | JGI | 06/2011 |
| Basidiomycota | Dsq | Dichomitus squalens | JGI | 06/2011 |
| Basidiomycota | Gsa | Gelatoporia subvermispora | JGI | 06/2011 |

| | | | | |
|---|---|---|---|---|
| Basidiomycota | Fpi | Fomitopsis pinicola | JGI | 06/2011 |
| Basidiomycota | Wco | Wolfiporia cocos | JGI | 06/2011 |
| Basidiomycota | Ppl | Postia placenta | JGI | 06/2011 |
| Basidiomycota | Fma | Fomitiporia mediterranea | JGI | 06/2011 |
| Basidiomycota | Tms | Tremella mesenterica | JGI | 06/2011 |
| Basidiomycota | Cne | Cryptococcus neoformans | JGI | 06/2011 |
| Basidiomycota | Pta | Puccinia triticina | Broad Institute | 06/2011 |
| Basidiomycota | Mlr | Melampsora larici-populina | JGI | 06/2011 |
| Basidiomycota | Rgr | Rhodotorula graminis | JGI | 06/2011 |
| Basidiomycota | Sro | Sporobolomyces roseus | JGI | 06/2011 |
| Basidiomycota | Mgl | Malassezia globosa | JGI | 06/2011 |
| Basidiomycota | Uma | Ustilago maydis | Broad Institute | 06/2011 |
| Basidiomycota | Wse | Wallemia sebi | JGI | 06/2011 |
| Taphrinomycotina | Soc | Schizosaccharomyces octosporus | Broad Institute | 06/2011 |
| Taphrinomycotina | Scr | Schizosaccharomyces cryophilus | Broad Institute | 06/2011 |
| Taphrinomycotina | Sja | Schizosaccharomyces japonicus | Broad Institute | 06/2011 |
| Saccharomycotina | Cdu | Candida dubliniensis | KEGG | 06/2011 |
| Saccharomycotina | Ctr | Candida tropicalis | Broad Institute | 06/2011 |
| Saccharomycotina | Cpp | Candida parapsilosis | Broad Institute | 06/2011 |
| Saccharomycotina | Lel | Lodderomyces elongisporus | JGI | 06/2011 |
| Saccharomycotina | Pst | Scheffersomyces stipitis | JGI | 06/2011 |
| Saccharomycotina | Dha | Debaryomyces hansenii | Genolevures | 06/2011 |
| Saccharomycotina | Mgd | Meyerozyma guilliermondii | JGI | 06/2011 |
| Saccharomycotina | Clu | Clavispora lusitaniae | Broad Institute | 06/2011 |
| Saccharomycotina | Ppa | Pichia pastoris | JGI | 06/2011 |
| Saccharomycotina | Spa | Saccharomyces paradoxus | The Hyphal Tip | 06/2011 |
| Saccharomycotina | Smi | Saccharomyces mikatae | The Hyphal Tip | 06/2011 |
| Saccharomycotina | Sku | Saccharomyces kudriavzevii | The Hyphal Tip | 06/2011 |
| Saccharomycotina | Sba | Saccharomyces bayanus | YGOB | 06/2011 |
| Saccharomycotina | Cgl | Candida glabrata | Genolevures | 06/2011 |
| Saccharomycotina | Sca | Naumovia castellii | YGOB | 06/2011 |
| Saccharomycotina | Kpo | Vanderwaltozyma polyspora | YGOB | 06/2011 |
| Saccharomycotina | Zro | Zygosaccharomyces rouxii | JGI | 06/2011 |
| Saccharomycotina | Kla | Kluyveromyces lactis | Genolevures | 06/2011 |
| Saccharomycotina | Ago | Ashbya gossypii | YGOB | 06/2011 |
| Saccharomycotina | Skl | Lachancea kluyveri | Genolevures | 06/2011 |
| Saccharomycotina | Kwa | Lachancea waltii | Duke | 06/2011 |
| Saccharomycotina | Lth | Lachancea thermotolerans | JGI | 06/2011 |
| Pezizomycotina | Nte | Neurospora tetrasperma | JGI | 06/2011 |

| | | | | |
|---|---|---|---|---|
| Pezizomycotina | Ndi | Neurospora discreta | JGI | 06/2011 |
| Pezizomycotina | Pan | Podospora anserina | JGI | 06/2011 |
| Pezizomycotina | Tts | Thielavia terrestris | JGI | 06/2011 |
| Pezizomycotina | Cgo | Chaetomium globosum | JGI | 06/2011 |
| Pezizomycotina | Mta | Myceliophthora thermophila | JGI | 06/2011 |
| Pezizomycotina | Cpr | Cryphonectria parasitica | JGI | 06/2011 |
| Pezizomycotina | Mor | Magnaporthe oryzae | JGI | 06/2011 |
| Pezizomycotina | Ggr | Glomerella graminicola | Broad Institute | 06/2011 |
| Pezizomycotina | Chg | Colletotrichum higginsianum | Broad Institute | 06/2011 |
| Pezizomycotina | Val | Verticillium albo-atrum | Broad Institute | 06/2011 |
| Pezizomycotina | Vda | Verticillium dahliae | Broad Institute | 06/2011 |
| Pezizomycotina | Tat | Trichoderma atroviride | JGI | 06/2011 |
| Pezizomycotina | Tvi | Hypocrea virens | JGI | 06/2011 |
| Pezizomycotina | Tre | Hypocrea jecorina | JGI | 06/2011 |
| Pezizomycotina | Nha | Nectria haematococca | JGI | 06/2011 |
| Pezizomycotina | Gze | Gibberella zeae | JGI | 06/2011 |
| Pezizomycotina | Fox | Fusarium oxysporum | Broad Institute | 06/2011 |
| Pezizomycotina | Fve | Gibberella moniliformis | Broad Institute | 06/2011 |
| Pezizomycotina | Bci | Botryotinia fuckeliana | JGI | 06/2011 |
| Pezizomycotina | Ssc | Sclerotinia sclerotiorum | JGI | 06/2011 |
| Pezizomycotina | Aco | Aspergillus carbonarius | JGI | 06/2011 |
| Pezizomycotina | Ang | Aspergillus niger | JGI | 06/2011 |
| Pezizomycotina | Ate | Aspergillus terreus | JGI | 06/2011 |
| Pezizomycotina | Afl | Aspergillus flavus | JGI | 06/2011 |
| Pezizomycotina | Aor | Aspergillus oryzae | JGI | 06/2011 |
| Pezizomycotina | Acl | Aspergillus clavatus | JGI | 06/2011 |
| Pezizomycotina | Afu | Aspergillus fumigatus | Broad Institute | 06/2011 |
| Pezizomycotina | Nfi | Neosartorya fischeri | JGI | 06/2011 |
| Pezizomycotina | Pcm | Penicillium chrysogenum | JGI | 06/2011 |
| Pezizomycotina | Pbr | Paracoccidioides brasiliensis | Broad Institute | 06/2011 |
| Pezizomycotina | Bdr | Ajellomyces dermatitidis | Broad Institute | 06/2011 |
| Pezizomycotina | Acs | Ajellomyces capsulatus | JGI | 06/2011 |
| Pezizomycotina | Ure | Uncinocarpus reesii | Broad Institute | 06/2011 |
| Pezizomycotina | Cim | Coccidioides immitis | Broad Institute | 06/2011 |
| Pezizomycotina | Cpd | Coccidioides posadasii | Broad Institute | 06/2011 |
| Pezizomycotina | Aot | Arthroderma otae | Broad Institute | 06/2011 |
| Pezizomycotina | Mgy | Microsporum gypseum | Broad Institute | 06/2011 |
| Pezizomycotina | Tto | Trichophyton tonsurans | Broad Institute | 06/2011 |
| Pezizomycotina | Teq | Trichophyton equinum | Broad Institute | 06/2011 |

| | | | | |
|---|---|---|---|---|
| Pezizomycotina | Trb | Trichophyton rubrum | Broad Institute | 06/2011 |
| Pezizomycotina | Tve | Trichophyton verrucosum | Broad Institute | 06/2011 |
| Pezizomycotina | Abe | Arthroderma benhamiae | Broad Institute | 06/2011 |
| Pezizomycotina | Mgm | Mycosphaerella graminicola | JGI | 06/2011 |
| Pezizomycotina | Mii | Mycosphaerella pini | JGI | 06/2011 |
| Pezizomycotina | Mfi | Mycosphaerella fijiensis | JGI | 06/2011 |
| Pezizomycotina | Mpo | Mycosphaerella populorum | JGI | 06/2011 |
| Pezizomycotina | Pno | Phaeosphaeria nodorum | JGI | 06/2011 |
| Pezizomycotina | Che | Cochliobolus heterostrophus | JGI | 06/2011 |
| Pezizomycotina | Pti | Pyrenophora tritici-repentis | Broad Institute | 06/2011 |
| Pezizomycotina | Abr | Alternaria brassicicola | JGI | 06/2011 |

# Table S5. Extended proteome data-set

| Taxonomic Group | Code | Scientific Species Name | Source | As of |
|---|---|---|---|---|
| Microsporidia | Ehe | Encephalitozoon hellem | GenBank | 10/2010 |
| Microsporidia | Oba | Octosporea bayeri | GenBank | 09/2009 |
| Microsporidia | Vcu | Vavraia culicis | GenBank | 03/2011 |
| Zygomycota | Mov | Mortierella verticillata | Broad Institute | 02/2011 |
| Saccharomycotina | Yli | Yarrowia lipolytica | Genolevures | 06/2011 |
| Pezizomycotina | Tme | Tuber melanosporum | KEGG | 06/2011 |
| Metazoa; Eumetazoa | CIOIN | Ciona intestinalis | Quest For Orthologs | 03/2011 |
| Metazoa; Eumetazoa | DANRE | Danio rerio | Quest For Orthologs | 08/2011 |
| Metazoa; Eumetazoa | DROME | Drosophila melanogaster | Quest For Orthologs | 08/2011 |
| Metazoa; Eumetazoa | HUMAN | Homo sapiens | Quest For Orthologs | 08/2011 |
| Rhodophyta | CYAME | Cyanidioschyzon merolae | BruceiDB | 10/2010 |
| Viridiplantae | ARATH | Arabidopsis thaliana | Quest For Orthologs | 08/2011 |
| Viridiplantae | CHLRE | Chlamydomonas reinhardtii | Phytozome v7.0 | 07/2011 |
| Viridiplantae | ORYSI | Oryza sativa subsp. indica | ENSEMBL - Plants | 07/2011 |
| Viridiplantae | POPTR | Populus trichocarpa | Phytozome v7.0 | 07/2011 |

## Table S6. *E. cuniculi* sequences for concatenated tree

| Accession | Gene names | Code | Length | Protein names |
|---|---|---|---|---|
| Q8SSM5 | ECU01_0370 | Ecu0000034 | 295 | Zuotin-related protein |
| Q8SWP5 | ECU01_0380 | Ecu0000035 | 310 | Putative uncharacterized protein ECU01_0380 |
| Q8SSL3 | ECU01_0780 | Ecu0000076 | 428 | Zinc finger protein |
| Q8SSK4 | ECU01_1000 | Ecu0000099 | 196 | CDP-diacylglycerol insitol-3-phosphatidyltransferase |
| Q8SSI4 | ECU02_0240 | Ecu0000183 | 408 | U3 snoRNA-associated RNP (18S rRNA production) |
| Q8SSC3 | ECU03_0305 | Ecu0000344 | 95 | Vacuolar ATP synthase subunit F |
| Q8SW60 | ECU03_0420 | Ecu0000355 | 858 | Similarity to yeast CDC68 |
| Q8SW56 | ECU03_0530 | Ecu0000366 | 176 | Putative uncharacterized protein ECU03_0530 |
| Q8SW15 | ECU03_1240 | Ecu0000434 | 317 | Similarity to HYPOTHETICAL protein YGW1_yeast |
| Q8SS81 | ECU03_1350 | Ecu0000445 | 355 | Glycylpeptide N-tetradecanoyltransferase |
| Q8SW06 | ECU03_1380 | Ecu0000448 | 347 | Similarity to pelota protein |
| Q8SS59 | ECU04_0460 | Ecu0000517 | 277 | Dimethyladenosine transferase |
| Q8SS40 | ECU04_0900 | Ecu0000561 | 557 | Phenylalanine-tRNA ligase beta chain |
| Q8SRV7 | ECU05_1120i | Ecu0000759 | 337 | Tyrosyl-tRNA ligase |
| Q8SRV0 | ECU05_1280 | Ecu0000775 | 567 | Double-strand break repair protein MRE11 |
| Q8SRU8 | ECU05_1320 | Ecu0000779 | 180 | U3 Small nucleolar ribonucleoprotein |
| Q8SVG2 | ECU05_1500 | Ecu0000797 | 435 | Similarity to HYPOTHETICAL WD-Repeat protein YER2_yeast |
| Q8SRT4 | ECU06_0200 | Ecu0000836 | 742 | DNA repair helicase of the RAD3/XPD subfamily |
| Q8SRS2 | ECU06_0360 | Ecu0000852 | 274 | Replication factor A protein 2 (32kDa subunit) |
| Q8SVD7 | ECU06_0420 | Ecu0000858 | 777 | Putative uncharacterized protein ECU06_0420 |
| Q8SV53 | ECU07_0080 | Ecu0000993 | 258 | Putative uncharacterized protein ECU07_0080 |
| Q8SRL9 | ECU07_0220 | Ecu0001007 | 291 | Transcription initiation factor TFIIE alpha subunit |
| Q8SRI0 | ECU07_1340 | Ecu0001119 | 573 | rRNA methyl-transferase |
| Q8SUV3 | ECU07_1630 | Ecu0001145 | 242 | Putative uncharacterized protein ECU07_1630 |
| Q8SRG9 | ECU07_1660 | Ecu0001148 | 475 | Probable phenylalanyl-tRNA synthetase alpha chain |

| | | | | |
|---|---|---|---|---|
| Q8SRG5 | ECU07_1750 | Ecu0001157 | 155 | DNA-directed RNA polymerase II 19kDa subunit |
| Q8SUS7 | ECU08_0440 | Ecu0001215 | 198 | Putative uncharacterized protein ECU08_0440 |
| Q8SRD5 | ECU08_0630 | Ecu0001234 | 298 | DNA primase |
| Q8SRC6 | ECU08_0760 | Ecu0001247 | 768 | Belongs to the XPF/ERCC4/RAD1 family |
| Q8SRB1 | ECU08_1100 | Ecu0001282 | 818 | Coatomer compex beta subunit |
| Q8SUJ3 | ECU08_1840 | Ecu0001353 | 247 | Putative uncharacterized protein ECU08_1840 |
| Q8SUI9 | ECU08_1900 | Ecu0001359 | 2832 | Putative uncharacterized protein ECU08_1900 |
| Q8STV7 | ECU09_0460 | Ecu0001428 | 218 | Putative leucine repeat-rich protein |
| Q8SQN2 | ECU09_0910 | Ecu0001474 | 362 | Deoxyhypusine synthase |
| Q8STR5 | ECU09_1010 | Ecu0001485 | 250 | Putative uncharacterized protein ECU09_1010 |
| Q8SQM7 | ECU09_1070 | Ecu0001491 | 278 | Putative peptidase |
| Q8SQM4 | ECU09_1210 | Ecu0001501 | 408 | GPI-anchor biosynthesis protein |
| Q8STQ0 | ECU09_1260 | Ecu0001506 | 326 | Putative uncharacterized protein ECU09_1260 |
| Q8STN3 | ECU09_1520 | Ecu0001534 | 179 | Putative uncharacterized protein ECU09_1520 |
| Q8STM7 | ECU09_1590 | Ecu0001541 | 206 | Similarity to HYPOTHETICAL transmembrane protein YNO3_YEAST |
| Q8SUH2 | ECU10_0280 | Ecu0001614 | 825 | Putative uncharacterized protein ECU10_0280 |
| Q8SR66 | ECU10_0380 | Ecu0001624 | 298 | mRNA cap guanine-N7 methyltransferase |
| Q8SR55 | ECU10_0600 | Ecu0001646 | 623 | DNA replication factor A protein 1 |
| Q8SUF0 | ECU10_0800 | Ecu0001666 | 210 | Putative uncharacterized protein ECU10_0800 |
| Q8SR32 | ECU10_1080 | Ecu0001693 | 418 | DNA polymerase alpha subunit B |
| Q8SR23 | ECU10_1210 | Ecu0001706 | 1799 | DNA polymerase |
| Q8SUC4 | ECU10_1340 | Ecu0001719 | 223 | Putative uncharacterized protein ECU10_1340 |
| Q8SU39 | ECU11_1070 | Ecu0001880 | 299 | Similarity to HYPOTHETICAL oxidoreductase YAEB_schpo |
| Q8SQU7 | ECU11_1310 | Ecu0001904 | 345 | NADPH adrenodoxin oxidoreductase |
| Q8SQT5 | ECU11_1490 | Ecu0001922 | 333 | Splicing factor for U2 snRNP |
| Q8SU19 | ECU11_1520 | Ecu0001925 | 243 | Similarity to HYPOTHETICAL methyltransferase-like protein YGGH_ecoli |
| Q8STZ8 | ECU11_1930 | Ecu0001966 | 571 | Similarity to putative transcription factor SPT5 |
| Q8SQR0 | ECU11_2000 | Ecu0001973 | 1025 | Putative nuclear protein of the SMC family |
